# Mesenchymal stem cell and exosome-based therapies for degenerative disc disease: from mechanisms to clinical translation

**DOI:** 10.3389/fcell.2026.1802828

**Published:** 2026-05-13

**Authors:** Baoliang Li, Yanyan Zhang, Changjiao Ji, Zhigang Shi, Nianhu Li

**Affiliations:** 1 Department of Orthopedics, Affiliated Hospital of Shandong University of Traditional Chinese Medicine, Jinan, China; 2 Department of Rheumatology and Immunology, Affiliated Hospital of Shandong University of Traditional Chinese Medicine, Jinan, China

**Keywords:** clinical translation, degenerative disc disease, exosomes, mesenchymal stem cells, regenerative therapy

## Abstract

Degenerative disc disease (DDD) is a leading cause of chronic low back pain and disability worldwide, placing a significant burden on public health systems and economies. The pathogenesis of DDD is characterized by oxidative stress, chronic inflammation, dysregulated cell death, and impaired extracellular matrix (ECM) homeostasis, all of which contribute to the structural degradation and functional impairment of intervertebral discs. Current clinical treatments primarily offer palliative relief, underscoring the urgent need for regenerative therapies that address the underlying pathological mechanisms. Mesenchymal stem cells (MSCs) and their exosomes (Exos) have emerged as promising candidates for DDD therapy, as they stimulate ECM synthesis, regulate inflammation, and enhance cell survival. Furthermore, advanced biomaterials have been developed to create bioactive environments that enhance cell retention and facilitate controlled therapeutic delivery. This review provides a comprehensive overview of the molecular mechanisms driving DDD, evaluates MSC/Exo and biomaterial-based therapies, and explores emerging technologies for personalized treatment strategies aimed at restoring disc function, extending beyond symptomatic management.

## 1 Introduction

Degenerative disc disease (DDD), a prevalent disorder characterized by the structural and functional deterioration of intervertebral discs (IVD) across all regions, is a leading cause of global low back pain (LBP) (van Uden et al., 2017; Wu et al., 2020). The Global Burden of Disease Study 2017 reported approximately 577 million LBP cases, with DDD prevalence peaking in adults aged 80–89 years and increasing with population aging (Manshaii and Tawil, 2023). Economically, DDD cost an estimated USD 26.05 billion in 2021, projected to reach USD 45.92 billion by 2029, disproportionately burdening low- and middle-income countries and reducing workforce productivity (de Oliveira et al., 2024).

The IVD is a critical avascular component of the spinal motion system, accounting for one-third of spinal height (Krut et al., 2021). It comprises three specialized regions: the central nucleus pulposus (NP), concentric annulus fibrosus (AF), and cartilaginous endplates (CEP) (Zhang et al., 2025a). The NP is a hydrophilic gel rich in proteoglycans, type II collagen, and 70% water, generating osmotic pressure for shock absorption. Notochordal cells in the developing NP are gradually replaced by chondrocyte-like cells with aging, linked to degeneration (Jin et al., 2025). The AF consists of lamellae: outer layers (predominantly type I collagen) provide tensile strength, while inner fibrocartilage (type II collagen, proteoglycans) resists compression. It acts as a physical barrier and suppresses NP vascularization. The CEP enables nutrient diffusion (>90% of disc needs) and anchors the IVD to vertebrae (Zhang et al., 2025a). Its ossification with aging reduces perfusion, a key degenerative trigger. The IVD’s unique microenvironment is defined by avascularity, hypoxia, acidosis, and high osmolarity (Rider et al., 2019). Nutrients and waste diffuse *via* the CEP and outer AF, but reduced endplate permeability during aging impairs this exchange (Wang et al., 2016). This harsh milieu limits cell survival and repair, rendering the IVD susceptible to permanent damage from minor pathological insults. Pathologically, DDD is marked by the gradual degeneration of the NP and AF, leading to diminished disc hydration and compromised structural integrity, ultimately resulting in chronic LBP, stiffness, and restricted mobility (Mohd Isa et al., 2022). While common in older adults, it also affects younger individuals with spinal trauma or genetic susceptibility (Choi et al., 2018). Advanced stages may cause nerve compression and severe pain. Current clinical treatments, including conservative management and invasive surgical interventions, offer only temporary symptom relief without addressing the root pathological causes of DDD. These treatments are further limited by high rates of symptom recurrence, surgical complications, long-term spinal function impairment, and inability to address the high clinical heterogeneity of the disease, driving intensive research toward biological regenerative therapies that can fundamentally overcome these clinical limitations (Beall et al., 2021).

Therapies based on mesenchymal stem cells (MSCs) have emerged as a promising regenerative strategy for DDD, with significant advancements in preclinical and clinical research. These cells possess dual therapeutic potential: they can differentiate into NP-like phenotypes to replenish the depleted resident cell pool and exert paracrine effects to modulate the pathological IVD microenvironment. Therefore, compared with conventional treatments that mainly target pain and disability, MSC-based therapies are being explored for their potential to restore biological function within the disc by promoting matrix synthesis, improving disc hydration, and suppressing inflammation- and apoptosis-driven degeneration. Combining MSCs with growth factors further enhances tissue regeneration and disc hydration, optimizing therapeutic outcomes (Kumar and Kumar, 2025; Pont et al., 2025). Moreover, MSC-derived exosomes (Exos) have gained attention as a cell-free alternative, offering improved stability and a reduced risk of immune rejection. Importantly, Exo-based approaches may retain many of the regenerative and immunomodulatory benefits of MSCs while reducing some practical concerns associated with live-cell transplantation, thereby providing a potentially safer and more standardized strategy for clinical translation. Their therapeutic effects are mediated by mitigating inflammation and promoting extracellular matrix (ECM) production in NP cells (Hu et al., 2023). Clinical trials have validated the efficacy of MSCs in alleviating pain and improving function. Furthermore, MSC-mediated modulation of key molecular pathways suppresses inflammation and apoptosis, potentially altering DDD progression (Zhu L. et al., 2020; Yang and Yang, 2024). However, critical barriers remain to clinical translation: the harsh IVD microenvironment impairs stem cell survival and function, alongside challenges in cell sourcing, delivery methods, patient variability, and ethical and regulatory compliance (Wang et al., 2025; Ikwuegbuenyi et al., 2025; Li et al., 2025). These advancements and remaining hurdles underscore the potential of MSC- and Exo-based therapies to move DDD management beyond symptom control toward biologically informed regeneration, while also highlighting the need for further research to address translational gaps, including delivery optimization, product standardization, and long-term efficacy validation.

This review summarizes recent advances and challenges in MSC- and Exo-based regenerative therapies for DDD. To bridge the gap between promising preclinical results and clinical application, it critically discusses the pathophysiology of DDD, the therapeutic mechanisms of MSCs and their derivatives, and current developments in cell-based and cell-free strategies. Furthermore, it addresses issues related to these techniques, including optimizing delivery methods and overcoming clinical translation challenges, providing a comprehensive outlook on the future of treating this debilitating condition.

## 2 Mechanisms of DDD

**FIGURE 1:**
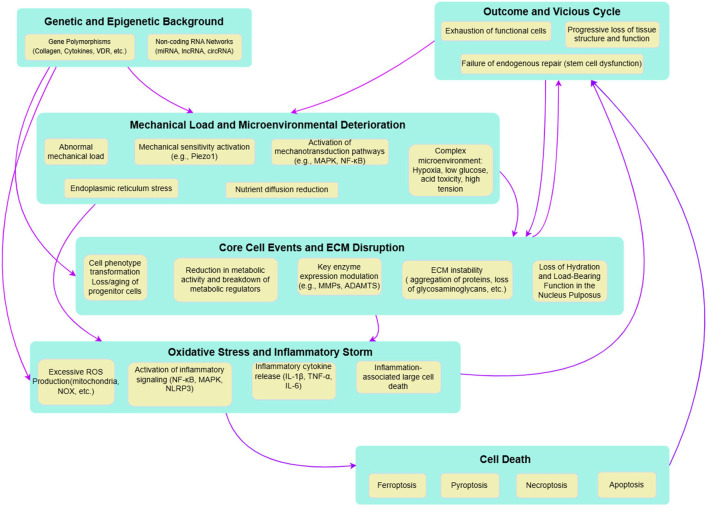
Depicts the intricate molecular mechanisms underlying DDD. The pathways shown highlight the interactions among genetic, mechanical, oxidative, and inflammatory factors, which collectively contribute to cellular dysfunction and DDD.

DDD is a multifactorial pathological process driven by the complex interaction of biochemical, mechanical, and genetic factors. At the molecular level, this degenerative cascade involves interactions between genetic predispositions, inflammatory mediators, and ECM remodeling enzymes (Xie et al., 2022). The avascular, hypoxic nature of the IVD creates a challenging microenvironment characterized by low pH, nutrient deprivation, and accumulation of metabolic waste, collectively driving the degenerative process and severely limiting tissue self-repair capabilities (Zhang S. et al., 2021) (Figure 1).

### 2.1 ECM homeostasis disruption and failure of endogenous repair

The primary pathological alteration in DDD is the disruption of ECM metabolic homeostasis. This disruption begins with a critical shift in cellular phenotype: the depletion of large vacuolated notochordal cells from the NP, replaced by functionally inferior chondrocyte-like cells of unclear origin (Sakai et al., 2012). Large vacuolated notochordal cells normally secrete factors, such as connective tissue growth factors, that stimulate cell proliferation and matrix synthesis. Their decline leads to a collapse in anabolic capacity, downregulation of notochordal markers, and upregulation of pro-inflammatory mediators, shifting the microenvironment toward a catabolic-dominant state (Erwin et al., 2006; Sinkemani et al., 2020).

Concurrently, the ECM’s components and structural integrity deteriorate. The collagen network shifts from predominantly type II to type I collagen, with a substantial decline in proteoglycan levels. Notably, aggrecan (ACAN)—responsible for core hydration—is significantly reduced (Mohd Isa et al., 2022). These compositional changes are accompanied by a surge in matrix-degrading enzyme activity. Matrix metalloproteinases (MMPs), specifically MMP-1, MMP-2, MMP-3, MMP-9, MMP-13, and MMP-14, are upregulated in degenerated discs and play pivotal roles in collagen degradation (Zou et al., 2023). Additionally, a disintegrin and metalloproteinase with thrombospondin motifs (ADAMTS) enzymes, including ADAMTS-4 and ADAMTS-5, primarily mediate ACAN cleavage and proteoglycan loss (Ogaili et al., 2025). Furthermore, the degradation of link proteins and core proteins in proteoglycan aggregates, such as versican, and alterations in small leucine-rich proteoglycans like decorin and biglycan, disrupt the ECM’s structural support and its interaction with the collagen network. These enzymatic changes result in a fundamental imbalance between ECM synthesis and degradation, severely compromising the disc’s structural integrity. The loss of proteoglycans, especially ACAN, reduces the NP’s hydration capacity, lowering hydrostatic pressure and impairing load-bearing function.

Matrix failure in DDD reflects not only enhanced catabolism but also impaired endogenous repair. Progenitor- or stem-like cell populations in degenerated discs show reduced chemotaxis, survival, proliferation and differentiation, thereby limiting intrinsic regeneration. In parallel, senescent disc cells accumulate and acquire a senescence-associated secretory phenotype (SASP), which amplifies inflammatory and catabolic signalling and further suppresses matrix anabolism (Patil et al., 2018). Together, ECM disequilibrium and loss of reparative capacity form a central pathological basis for DDD and help explain why structural restoration becomes increasingly difficult once degeneration is established.

### 2.2 Oxidative stress, inflammatory signaling, and interplay with novel cell death mechanisms

Oxidative stress is increasingly recognised as a key driver of DDD progression and as a mechanistic bridge linking inflammation, ECM breakdown and loss of viable disc cells. Reactive oxygen species (ROS), generated through mitochondrial dysfunction, NADPH oxidases and related processes, disrupt redox homeostasis, damage lipids and nucleic acids, impair ATP production and sensitise disc cells to inflammatory and catabolic stimuli (Ma et al., 2025; Shi et al., 2023). These effects are closely integrated with inflammatory signalling networks, among which NF-κB is one of the best characterised. Cytokines such as IL-1β and TNF-α activate NF-κB and induce expression of MMPs, ADAMTS enzymes, chemokines and other inflammatory mediators, thereby coupling inflammation directly to matrix destruction (Sun et al., 2015). MAPK signalling, including p38, JNK and ERK, further integrates inflammatory and mechanical cues, whereas PI3K–Akt, Wnt–β-catenin and Piezo1-associated signalling appear to modulate survival, stress adaptation and maladaptive remodelling in a context-dependent manner (Du et al., 2023; Zhang et al., 2025b). Oxidative injury is also linked to non-enzymatic matrix modification. Advanced glycation end products (AGEs), including pentosidine and carboxymethyllysine, accumulate in disc tissue with ageing and metabolic stress (Meiliana et al., 2018). Overall, AGE accumulation is associated with impaired matrix quality, altered tissue mechanics and degenerative remodelling, rather than simply with bulk matrix loss.

In addition to apoptosis, DDD involves multiple forms of regulated cell death. Pyroptosis and ferroptosis have attracted particular attention because both may reduce the viable disc cell pool while intensifying inflammatory or oxidative injury. Pyroptosis is commonly associated with activation of the NLRP3 inflammasome, caspase-1 activation and release of IL-1β and IL-18, thereby linking inflammatory signalling to membrane rupture and tissue damage (Luo et al., 2023). Ferroptosis is an iron-dependent form of regulated cell death driven by lipid peroxidation and associated with altered antioxidant defence, including GPX4-and NRF2-related pathways (Jin et al., 2025). Autophagy appears more context-dependent: basal autophagy may support homeostasis, whereas dysregulated or sustained autophagy can accompany chronic cellular stress. Necroptosis and other non-apoptotic programmes may also contribute, although their relative importance in human DDD remains less clear (Hao et al., 2022).

From a regenerative perspective, however, these mechanisms are unlikely to represent equivalent therapeutic targets. The most consequential pathways are likely to be those that determine whether a permissive reparative niche can be re-established within the degenerated disc. In this context, the ROS–NF-κB inflammatory axis is likely to represent the highest-priority target because it functions as an upstream hub linking cytokine amplification, matrix catabolism, senescence and loss of reparative cell fitness (Ma et al., 2025; Shi et al., 2023; Sun et al., 2015). By contrast, NLRP3 inflammasome-mediated pyroptosis and ferroptosis are better regarded as downstream effector mechanisms. Although these pathways are clearly relevant because they directly deplete resident disc cells and may compromise transplanted MSCs, targeting them alone is unlikely to provide durable benefit if the broader inflammatory and metabolic microenvironment remains uncorrected (Jin et al., 2025; Luo et al., 2023). MAPK signalling may similarly act more as a stress-responsive amplifier than as a singular master node (Du et al., 2023). Thus, effective regenerative strategies will probably need first to attenuate the ROS–NF-κB-driven hostile niche and then, where appropriate, incorporate more specific anti-pyroptotic or anti-ferroptotic interventions.

### 2.3 Biomechanical stress and deterioration of the disc microenvironment

Aberrant mechanical loading is another major determinant of DDD and acts in close reciprocity with molecular degeneration. Physiological dynamic loading is required for disc nutrition and matrix homeostasis, whereas static overload, repetitive trauma and non-physiological stress patterns induce inflammatory signalling, ECM catabolism and cell injury. Mechanotransduction in this setting involves integrin-associated pathways, focal adhesion signalling, MAPK activation and mechanosensitive ion channels such as Piezo1, which together influence calcium influx, mitochondrial homeostasis, inflammatory mediator production, senescence and matrix remodelling (Xia et al., 2024).

These effects are compounded by progressive deterioration of the disc microenvironment. The CEP is the principal route for nutrient influx and metabolite efflux in the inner disc. CEP calcification, reduced porosity and impaired permeability hinder solute diffusion and are associated with reduced glucose and oxygen availability, as well as lower pH, in the NP and inner AF (Ishihara and Urban, 1999). This is particularly important because the IVD already functions close to the limits of metabolic sufficiency under physiological conditions. Even modest impairment of endplate transport may therefore reduce matrix synthesis, promote acidification and constrain the reparative potential of resident or transplanted cells. In this setting, it is more precise to describe an altered osmotic milieu and loss of osmotic swelling pressure than a simple state of hyperosmolality, as osmotic regulation in the NP is dynamic and closely linked to aggrecan content and loading conditions.

These metabolic and biomechanical constraints have direct translational relevance. A degenerated disc niche characterised by acidity, poor nutrient supply, inflammatory activation and altered matrix mechanics is less permissive for cell survival, migration, differentiation and functional paracrine signalling. This may help explain why regenerative interventions can show biological activity yet fail to achieve durable repair when the host environment remains unfavourable. Restoration of microenvironmental permissiveness—particularly improvement of CEP-mediated nutrient transport and mitigation of hostile metabolic stress—should therefore be considered a second therapeutic priority alongside suppression of the ROS–NF-κB inflammatory axis (Ishihara and Urban, 1999; Lai et al., 2008). Thus, the disc microenvironment is not merely a background feature of disease progression, but a central determinant of whether matrix restoration and cell-based repair can translate into sustained regeneration.

### 2.4 Genetic susceptibility and ncRNAs

Genetic background contributes to inter-individual variability in DDD susceptibility and progression, although the strength and reproducibility of individual associations vary across populations. Reported contributors include polymorphisms in genes related to collagens, proteoglycans, ECM-remodelling enzymes, inflammatory mediators and vitamin D receptor signalling (Trefilova et al., 2021; Biczo et al., 2020). Experimental studies further suggest that vitamin D insufficiency may aggravate disc degeneration by perturbing inflammatory and metabolic homeostasis, although the relevance of these mechanisms to human DDD remains to be fully defined (Yang et al., 2025).

Beyond inherited variants, ncRNAs have emerged as important post-transcriptional regulators of DDD-related pathways. miRNAs, lncRNAs and circRNAs can modulate ECM metabolism, inflammation, senescence and regulated cell death through complex regulatory networks. For example, lncRNA MIR155HG has been linked to NP cell pyroptosis through the miR-223-3p–NLRP3 axis, whereas circ_0072464 has been reported to regulate ferroptosis through a miR-431–NRF2-dependent mechanism in preclinical models (Jin et al., 2025; Chen S. et al., 2025). At present, the translational value of these pathways may lie less in their immediate use as universal therapeutic targets than in their potential for mechanistic stratification, biomarker development and future precision approaches to DDD.

Collectively, the mechanisms of DDD are better organised as a therapeutic hierarchy than as a flat list of parallel pathways. Disruption of ECM homeostasis and impaired endogenous repair form the core structural and cellular basis of degeneration. From the perspective of regenerative therapy, however, the most consequential upstream barriers are the ROS-driven inflammatory microenvironment—within which NF-κB acts as a major integrative hub—and deterioration of disc nutritional and metabolic support caused by CEP dysfunction and impaired solute transport (Ma et al., 2025; Shi et al., 2023; Sun et al., 2015; Ishihara and Urban, 1999; Lai et al., 2008). These processes not only accelerate matrix catabolism and cellular dysfunction, but also determine whether endogenous progenitors, transplanted MSCs or exogenously delivered Exos can survive and function within the degenerated niche. In this framework, pyroptosis and ferroptosis are best viewed as important downstream effectors that exacerbate cell loss and regenerative failure, and thus as adjunctive rather than sufficient stand-alone targets (Jin et al., 2025; Du et al., 2023). Genetic susceptibility and ncRNA-mediated regulation further shape inter-individual heterogeneity in disease behaviour and therapeutic responsiveness (Yang et al., 2025). This hierarchical view provides a stronger mechanistic rationale for MSC- and Exo-based therapies: durable regeneration will probably require not only enhancement of matrix anabolism, but also coordinated modulation of the inflammatory–oxidative niche, preservation of reparative cell viability and at least partial restoration of disc microenvironmental permissiveness.

## 3 MSC therapies for DDD: therapeutic mechanisms and cell source optimization

**FIGURE 2:**
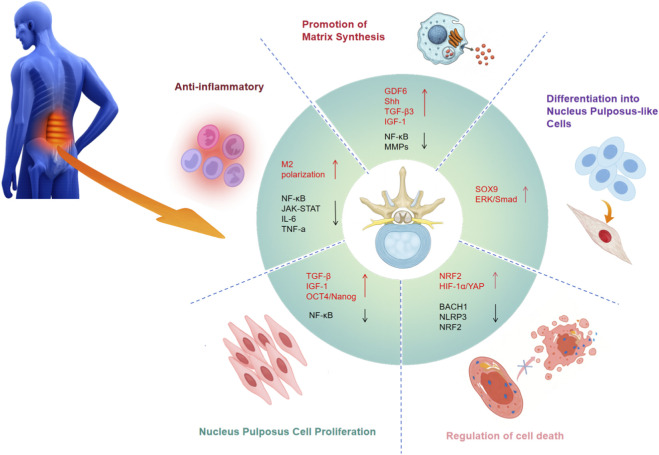
Demonstrates the mechanisms through which MSCs aid in the regeneration of the NP in DDD. MSCs are integral in modulating inflammation, enhancing matrix synthesis, and facilitating the differentiation of NP-like cells.

MSC-based therapy offers a promising regenerative approach for DDD, operating through synergistic mechanisms to restore NP structure and function, reduce inflammation, and promote tissue repair. Optimized MSC sources further enhance therapeutic outcomes by improving cell survival, differentiation, and adaptability to the challenging disc microenvironment. These interconnected mechanisms collectively reverse the degenerative cascade, with functional ECM synthesis as a key step, as matrix loss and impaired mechanical properties are central drivers of DDD progression. The following sections describe how MSCs regulate matrix synthesis to restore a physiologically competent NP microenvironment, followed by a discussion of other core therapeutic mechanisms and the strategic optimization of MSC sources for clinical application (Figure 2).

### 3.1 Promotion of matrix synthesis

In MSC-based therapy for DDD, promoting matrix synthesis and regulating its mechanical properties are critical for restoring NP function and integrity. Extensive studies have shown that MSCs, under specific inductive cues, can significantly enhance the synthesis of key ECM components—particularly proteoglycans and type II collagen (COL2A1)—thereby improving matrix hydrophilicity and elasticity. For example, the application of notochord-conditioned medium (NCCM) has been shown to stimulate human MSCs to differentiate into a young NP phenotype during pellet culture. This significantly increases the production of sulfated glycosaminoglycan (GAG) and upregulates genes associated with early IVD development, such as type III collagen and laminin β1 (Li et al., 2018; Korecki et al., 2010). This NCCM-induced phenotype is distinct from the traditional TGF-β-induced chondrogenic phenotype, closely resembling developing NP tissue, and allows MSCs to reconstruct a gel-like matrix with high water retention. Moreover, precise modulation of signaling pathways can further optimize differentiation efficiency. Research has demonstrated that small-molecule smoothened agonists can activate the Sonic Hedgehog (Shh) signaling pathway, which, when combined with TGF-β3, synergistically promotes the differentiation of adipose-derived MSCs (ADMSCs) into NP-specific cells (Hua et al., 2019). This synergy is reflected in the significant upregulation of NP-specific markers, increased expression of ACAN, SOX9, and COL2A1 genes and proteins, and enhanced GAG synthesis, thereby more accurately simulating the biochemical composition and matrix characteristics of a healthy NP.

MSCs also exert significant regulatory effects on degenerated NP cells through paracrine mechanisms. Co-culture studies have shown that MSCs not only upregulate their own ECM-related genes (e.g., ACAN, VCAN, SOX9, COL2A1, COL6A1) but also significantly boost the collagen biosynthetic capacity of degenerated NP cells. Simultaneously, MSCs suppress the production of pro-inflammatory cytokines (including IL-1α, IL-1β, IL-6, and TNF-α), improving the local inflammatory microenvironment and creating a favorable foundation for ECM deposition and remodeling. Additionally, MSCs secrete various growth factors (IGF-1, OP-1/BMP-7, GDF-7/BMP-12) in co-culture systems, further promoting cell proliferation, differentiation, and matrix synthesis, thus establishing a positive regulatory loop (Shim et al., 2016; Hu et al., 2015).

The mechanical strength of the matrix depends not only on its biochemical composition but also on its microstructure and the mechanical environment of the cells. Matrix stiffness, a critical physical signal, directly influences the differentiation fate and matrix remodeling ability of MSCs and NP-derived stem cells (NP-SCs). Studies using Tetronic-fibrinogen hydrogels with adjustable stiffness have revealed that when matrix stiffness simulates the physiological stiffness of a healthy NP (approximately 1 kPa), encapsulated NP-SCs exhibit higher cell viability, proliferative capacity, and chondrogenic differentiation, as evidenced by upregulation of ACAN and SOX9 gene expression and substantial sGAG accumulation. In contrast, in stiffer matrices (2 kPa), NP-SCs tend to differentiate towards an osteogenic phenotype, expressing osteocalcin and osteopontin, which hinders functional NP matrix regeneration (Navaro et al., 2015). In addition to regulating physical properties, the selection of growth factors is crucial. Research has shown that growth differentiation factor 6 (GDF6) is more effective than TGF-β or GDF5 in inducing MSCs, especially ADMSCs, to differentiate into NP-like cells (Ohnishi et al., 2023). Under GDF6 stimulation, ADMSCs exhibit the highest levels of ACAN gene expression, the highest ACAN/COL2A1 gene ratio, and the highest synthesis of total sGAGs, forming more homogeneous matrix deposits (Clarke et al., 2014). Scanning acoustic microscopy further confirmed that GDF6-induced matrices exhibit mechanical properties more similar to natural, proteoglycan-rich NP tissue, whereas TGF-β tends to form matrices resembling articular cartilage.

Furthermore, regulating inflammatory states is crucial for maintaining matrix strength. Studies have shown that pro-inflammatory cytokines (e.g., TNF-α) activate the NF-κB pathway, which upregulates the synthesis of matrix-degrading enzymes, such as MMP-13 and ADAMTS-5, and induces the degradation of ACAN and COL2A1, leading to ECM structural deterioration. However, MSCs and their conditioned media can effectively inhibit NF-κB activation, reduce pro-inflammatory factor production, and indirectly protect matrix integrity, thereby maintaining its mechanical properties. For instance, conditioned medium from dental pulp stem cells (DPSCs) inhibits TNF-α-induced activation of the NF-κB and JAK-STAT pathways, reduces inflammatory factors like IL-6, and promotes COL2A1 generation, preserving matrix structure and function in inflammatory environments (Dong et al., 2022). The choice of cell source and culture system also significantly affects matrix characteristics. Research comparing bone marrow-derived MSCs (BMSCs) and ADMSCs in three-dimensional (3D) culture systems shows that ADMSCs have a greater potential to differentiate into NP-like cells, producing higher levels of GAGs and proteoglycans, and expressing superior markers (e.g., HIF-1α, GLUT1, SOX9, ACAN, COL2A1) (Dai et al., 2021).

Thus, MSCs respond to growth factors, signaling pathways, epigenetic regulation, and mechanical microenvironments, actively promoting the synthesis of matrix components with physiological activity. They also enhance matrix mechanical strength, resist degradation, and maintain tissue homeostasis (Wang et al., 2021). Combining optimized cell sources, appropriate inductive factors, simulated physiological mechanical stimulation, and 3D culture systems is expected to generate engineered tissues. These tissues will closely mimic natural NP in biochemical composition and mechanical properties, providing a solid foundation for structural repair and functional regeneration in DDD.

### 3.2 Anti-inflammatory and immunomodulatory effects

In the treatment of DDD, the anti-inflammatory and immunomodulatory effects of MSCs are important mechanisms. Studies show that MSCs regulate various inflammatory factors and immune cells *via* paracrine pathways, improving the microenvironment of the degenerated disc. For example, one study demonstrated that DPSCs abrogate TNF-α-induced activation of the NF-κB pathway, suppressing the production of pro-inflammatory factors such as IL-6 and CCL-7, and downregulating JAK-STAT pathway activity (Dong et al., 2022). This alleviated the inflammatory response in NP cells and inhibited their apoptosis. Moreover, DPSCs promoted the polarization of local M2 macrophages, further enhancing the anti-inflammatory effect and restoring immune balance in the disc.

Beyond directly inhibiting inflammatory signaling pathways, MSCs exert broad immunomodulatory effects by regulating immune cell function. For instance, in a disc degeneration model, conditioned medium from ADMSCs significantly reduced the expression of inflammatory factors such as IL-1β, IL-6, IL-17, and TNF-α induced by TNF-α stimulation, demonstrating strong immunosuppressive potential (Dong et al., 2022). Another study showed that co-culturing MSCs with NP cells not only downregulated pro-inflammatory gene expression in NP cells but also promoted ECM gene expression, such as COL2A1 and ACAN, *via* factors like TGF-β, thereby enhancing tissue repair while suppressing inflammation (Lehmann et al., 2018). These effects suggest that MSCs not only intervene directly in inflammatory signaling but also modulate the local immune environment, indirectly influencing the survival and function of disc cells.

Further mechanistic studies reveal that the immunoregulatory function of MSCs is closely linked to their regulation of the complement system and T-cell responses. In a fibrocartilage organ culture model, MSCs and their secretome inhibited complement system activation, reducing membrane attack complex formation and alleviating tissue damage and inflammation (Wu et al., 2024). MSCs also regulate T-cell proliferation and NK cell activity to maintain immune tolerance, preventing excessive immune responses from damaging disc tissue (Li et al., 2023). These multi-layered immunomodulatory mechanisms collectively define the core role of MSCs in treating DDD, alleviating inflammation-driven pathology and creating a favorable microenvironment for tissue regeneration.

### 3.3 Induced differentiation into NP-like cells

The differentiation of MSCs into NP-like cells is a mechanism underlying their potential for DDD repair. This process is regulated by transcription factors, signaling pathways, and the cellular microenvironment. Numerous studies have shown that overexpressing specific transcription factors can effectively guide this differentiation. For example, Khalid et al. used adenovirus-mediated overexpression of Sox-9 and Six-1 genes to induce chondrocyte precursor differentiation of umbilical cord-derived MSCs (UCMSCs), observing disc regenerative effects in a rat model of disc degeneration (Ekram et al., 2021). These factors worked synergistically to enhance the expression of critical chondrogenic genes, such as BMP2 and TGFβ1. Additionally, ncRNAs play a crucial role; for instance, Zhi et al. demonstrated that miR-365, by inhibiting HOXA9, relieved its transcriptional repression on HIF-1α, driving MSC differentiation into NP-like cells (Zhi et al., 2025). In addition to genetic regulation, extracellular signaling pathways are essential. Niu et al. demonstrated that MSCs can be induced to differentiate NP-like cells in the presence of TGF-β1, with significant upregulation of NP-specific phenotypic markers—including COL2A1, ACAN, SOX9, KRT19, and PAX1. This differentiation process is mediated by the activation of the p-ERK1/2 and p-Smad2/3 signaling pathways, highlighting the core role of MSCs in NP regeneration and the importance of pathway crosstalk in driving their commitment to the NP lineage (Niu et al., 2021).

The efficiency of induced differentiation depends on both biochemical signals and the physical microenvironment. Traditional two-dimensional cultures fail to replicate the *in vivo* NP structure, while 3D culture systems offer more physiologically relevant support. Navarro et al. provided evidence that a 3D hydrogel substrate mimicking the stiffness of healthy NP tissue (∼1 kPa) strongly induces chondrogenic differentiation of encapsulated NP-derived stem cells. This biomimetic matrix upregulated chondrogenic markers, such as ACAN and Sox9, and promoted GAG accumulation, emphasizing the role of matrix stiffness in guiding NP stem cell fate (Navaro et al., 2015). In contrast, stiffer environments induced osteogenic differentiation, highlighting the crucial role of mechanical properties in cell fate determination. Hypoxic conditions, another core feature of the NP, are also essential for differentiation induction. Sinkemani et al. demonstrated that culturing MSCs in NP cell-conditioned medium under hypoxic conditions upregulated typical NP markers, such as HIF-1α, ACAN, and collagen II, promoting the generation of functional NP-like cells (Sinkemani et al., 2020).

In selecting optimal cellular sources for regenerative applications, evidence has shown distinct differentiation propensities among MSCs from different tissues, guiding the rational optimization of cell-based therapies. Hodgkinson et al. compared BMSCs and ADMSCs, showing that ADMSCs exhibited robust activation of the SMAD1/5/8 and ERK1/2 signaling pathways in response to growth differentiation factor 6. This enhanced activity was attributed to higher expression of BMP receptor type 2 in ADMSCs, which increased proteoglycan and COL2A1 biosynthesis (Hodgkinson et al., 2020). Dai et al. further validated these findings by comparing the 2 cell populations in a 3D culture system mimicking the native NP microenvironment. Their results confirmed that ADMSCs exhibited elevated expression of HIF-1α, SOX9, and ACAN, along with increased GAG accumulation following induction (Dai et al., 2021). Collectively, these findings suggest that ADMSCs may have inherent advantages over BMSCs in differentiating into functional NP-like cells.

In summary, by integrating genetic regulation, signaling pathway activation, biomimetic 3D cultures, and selecting optimal cell sources, MSCs can be efficiently induced to differentiate into NP-like cells capable of synthesizing healthy ECM components, providing a strong cellular foundation for IVD regeneration.

### 3.4 Promotion of NP cell proliferation

The ability of MSCs to promote NP cell proliferation is attributed to their paracrine activity. Shim et al. demonstrated that both NP cells and degenerative disc cells showed significantly higher proliferation rates when indirectly co-cultured with MSCs or cultured in MSC-conditioned medium, compared to monolayer controls (Shim et al., 2016). This proliferative effect was mutual, as MSCs also exhibited enhanced proliferation when cultured in NP cell-conditioned medium, indicating a trophic interaction mediated by soluble factors. These findings redefine the role of MSCs from potential differentiation vehicles to active “biocatalysts,” expanding the pool of host cells, including endogenous progenitors and functional cells, and providing a cellular foundation for repair.

In addressing the functional decline of endogenous stem cells in degenerative conditions, studies have shown that the MSC secretome can effectively rejuvenate degenerated NP progenitor cells (D-NPSCs), with restoration of proliferative capacity being a key aspect. Zeng et al. provided direct evidence of this: treatment of D-NPSCs with conditioned medium from UCMSCs significantly enhanced their proliferative capacity, as determined by DNA content analysis and cell cycle assays (Zeng et al., 2020). This recovery in proliferation was accompanied by the upregulation of pluripotency markers (OCT4, Nanog) and MSC surface markers (CD29, CD105), suggesting that MSC-secreted factors not only stimulate cell cycle progression but also restore self-renewal capability by reprogramming cells away from a senescent or quiescent state, providing a sufficient pool of functional cells for tissue regeneration.

While paracrine signaling is fundamental, direct cell-cell contact creates a highly activated microenvironment that amplifies proliferation cues. Research indicates that direct contact between MSCs and NP cells leads to a greater upregulation of anabolic gene expression than non-contact co-culture systems, inducing a richer profile of pro-proliferative growth factors from MSCs, such as TGF-β and IGF-1 (Ruan et al., 2012; Cao et al., 2015). At the molecular pathway level, MSC-NP co-culture results in the upregulation of TGF-β and the downregulation of NF-κB, a master regulator of inflammation and catabolism, creating a more favorable biochemical milieu for cell survival and proliferation. Additionally, the physical properties of the matrix play a critical regulatory role. Studies show that NP-derived stem cells exhibit higher proliferative activity when cultured in 3D matrices mimicking the softness of healthy NP tissue, compared to stiffer matrices. This implies that MSC efficacy is dependent on a permissive mechanical microenvironment that transmits “pro-proliferation” signals *via* mechanosensors like integrins, working in concert with MSC-derived biochemical signals to form an integrated network governing cell fate (Navaro et al., 2015).

### 3.5 Regulation of cell death

In MSC-based therapies for DDD, modulating cell death pathways is a critical therapeutic target. MSCs protect against DDD progression by regulating various forms of NP cell death—including apoptosis, pyroptosis, and ferroptosis—through the coordinated modulation of multiple signaling pathways. For instance, conditioned medium from human DPSCs has been shown to inhibit TNF-α-induced activation of the NF-κB pathway, suppressing NP cell apoptosis and attenuating caspase-3 activation (Dong et al., 2022). This mechanism promotes NP cell survival and preserves cytoskeletal integrity under inflammatory stress. In another study, BMSCs were found to inhibit the mitochondrial apoptotic pathway under hypoxic conditions by activating the HIF-1α/YAP signaling axis. This adaptive response enhances BMSC survival in the harsh microenvironment of degenerated IVDs, suggesting that hypoxic preconditioning may improve the stress tolerance of transplanted MSCs (Wang et al., 2020).

Beyond apoptosis, pyroptosis—a recently identified form of inflammatory cell death—has been implicated in DDD pathogenesis. Evidence shows that MSCs and their secreted extracellular vesicles can inhibit the caspase-1/gasdermin D (GSDMD)-mediated pyroptotic pathway by targeting NLRP3 inflammasome activity. For example, UCMSC-derived Exos alleviate NP cell pyroptosis *via* the miR-26a-5p/METTL14/NLRP3 regulatory axis (Yang et al., 2024). Additionally, BMSCs delivered through hydrogel-based systems exert anti-pyroptotic effects on AF cells by reducing NLRP3 inflammasome activation. This occurs through sirtuin 1-mediated deacetylation of apoptosis-associated speck-like protein containing a CARD, mitigating oxidative stress-induced pyroptosis and ameliorating disc degeneration (Chen S. et al., 2025). These findings highlight MSCs’ multi-targeted capacity to modulate distinct programmed cell death pathways in degenerative discs.

In the oxidative stress-dominated microenvironment of degenerated IVDs, transplanted BMSCs are highly susceptible to ferroptosis, an iron-dependent, lipid peroxidation-driven cell death form. Ferroptosis is a major barrier to BMSC-based therapy efficacy (Xu et al., 2023). Overexpression of Prominin-2 has been shown to enhance BMSC resistance to ferroptosis by promoting F-box only protein 22 (FBXO22)-mediated ubiquitination and degradation of BTB domain and CNC homolog 1 (BACH1), thereby activating the NRF2 antioxidant signaling pathway. In preclinical models, combining Prominin-2-overexpressing BMSCs with TBE56—a small molecule enhancing Prominin-2’s anti-ferroptotic activity—improved IVD histological scores and significantly promoted NP cell proliferation (Xu et al., 2025). These findings demonstrate that MSCs not only interfere with aberrant cell death processes in degenerated discs but also enhance the survival of both transplanted stem cells and resident disc cells by modulating key molecular targets. These insights provide a solid foundation for optimizing MSC-based regenerative strategies for DDD repair.

### 3.6 Clinical-oriented analysis of MSC sources for DDD therapy

MSCs can be derived from various sources, including bone marrow, adipose tissue, and umbilical cord, each offering distinct advantages and limitations in clinical applications for DDD treatment. The IVD presents a harsh microenvironment characterized by hypoxia, nutrient deficiency, low pH, and mechanical stress, which significantly affect the survival and function of transplanted cells. Therefore, selecting an MSC source must consider not only differentiation potential and immunomodulatory capacity but also resilience to these degenerative conditions.

BMSCs are the most extensively studied and considered the gold standard for MSC-based therapies. They are known for their robust immunomodulatory effects, effectively inhibiting T-cell proliferation and modulating inflammatory responses, making them valuable for treating autoimmune and inflammatory conditions (Heo et al., 2016). BMSCs also exhibit high chondrogenic potential, particularly in 3D pellet cultures supplemented with growth factors like TGF-β1 and BMP-2. In equine models, BMSCs synthesized superior ECM quality, with a more homogeneous distribution of COL2A1 compared to other MSC sources, despite lower absolute collagen II production in some cases (Contentin et al., 2020). However, harvesting BMSCs is invasive, requiring iliac crest aspiration, which is associated with significant donor site morbidity and yields a low cell number (∼0.001–0.01% of nucleated cells) (Mohamed-Ahmed et al., 2021). Additionally, BMSC functionality declines with donor age, which is a critical consideration for clinical translation. Regarding environmental tolerance, BMSCs demonstrate moderate adaptability to hypoxia. Studies show that while their chondrogenesis improves under mild hypoxia, they may still be vulnerable to the severe hypoxia and acidosis present in degenerated discs (Adesida et al., 2012).

ADMSCs are obtained through minimally invasive procedures like liposuction, offering practical advantages such as high cell yields (∼2% of the stromal vascular fraction) and minimal donor site morbidity. They have strong proliferative capabilities and potent immunomodulatory functions, effectively suppressing immune responses in inflammatory environments (Maria et al., 2017). However, their chondrogenic potential is generally considered variable and inferior to that of BMSCs *in vitro*. Comparative studies in canines show that ADMSCs exhibit lower GAG deposition and fail to produce collagen type II under standard chondrogenic conditions, even with high doses of BMP-2 or BMP-6 supplementation (Teunissen et al., 2021). This suggests that ADMSCs may require optimized differentiation protocols for effective cartilage and disc regeneration. A key advantage of ADMSCs is their high tolerance to hypoxic stress, making them particularly suitable for surviving and functioning in the harsh, avascular environment of the degenerated IVD.

UCMSCs represent a promising allogeneic source, harvested from medical waste (the umbilical cord), thus avoiding donor morbidity and ethical concerns. It is important to distinguish between two primary sources: umbilical cord blood (UCB-MSCs) and the umbilical cord matrix or Wharton’s Jelly (UCM-MSCs). UCB-MSCs exhibit superior proliferative capacity and high chondrogenic potential, producing a dense, hyaline-like cartilage matrix when stimulated with BMP-2 and TGF-β1. They also possess strong immunomodulatory properties (Gregoire et al., 2019). In contrast, UCM-MSCs, while also exhibiting high proliferative rates, show poor chondrogenic potential *in vitro*. As demonstrated by Islam et al., UCMSCs failed to form a relevant cartilage matrix even after chondrogenic induction with various potent growth factor combinations, as evidenced by weak alcian blue staining and low collagen type II immunostaining compared to synovial membrane or Hoffa’s fat pad-derived MSCs (Islam et al., 2016; Islam, 2018). Furthermore, research on dedifferentiation-reprogrammed MSCs (De-MSCs) suggests a strategy for enhancing therapeutic potential. As explored by Yang et al., MSCs that undergo differentiation and subsequent dedifferentiation acquire enhanced properties, such as improved survival, migration, and anti-apoptotic capacities, which could be beneficial for enduring the degenerative disc environment. UCMSCs, with their “younger” biological state, combined with such innovative priming strategies, could potentially overcome limitations in chondrogenic differentiation for DDD therapy (Zhu et al., 2023).

The choice of MSC source for DDD therapy is multifaceted. BMSCs offer proven chondrogenic quality and immunomodulation but are limited by harvesting constraints and age-related decline. ADMSCs present a practical option with easy access, high yield, and exceptional tolerance to hypoxic stress, a critical advantage for the disc niche, although their chondrogenic output may require further optimization. UCMSCs provide a potent, “young” cell source with high expansion capacity, but their efficacy is source-dependent: UCB-MSCs show strong chondrogenic and immunomodulatory potential, whereas UCM-MSCs, despite poor innate chondrogenesis, exhibit high plasticity and can be effectively primed *via* preconditioning or dedifferentiation to enhance survival and immunomodulatory functions. Ultimately, the selection should align with therapeutic goals and the stage of disc degeneration. For robust matrix regeneration in moderately degenerated discs, BMSCs or UCB-MSCs may be preferred. For severely degenerated discs, where cell survival and immunomodulation are crucial, ADMSCs or preconditioned UCMSCs may be optimal candidates. Future research should focus on standardizing and optimizing preconditioning strategies to maximize the therapeutic potential of each MSC source for clinical translation in DDD.

### 3.7 Alternative stem cell sources for DDD

MSCs are the most extensively studied and clinically advanced cell type for IVD regeneration in DDD. Their widespread adoption stems from four well-documented strengths: easy accessibility, low immunogenicity, multilineage differentiation potential, and robust paracrine effects (Zhao et al., 2024). Despite these merits, MSCs have inherent limitations that curb their therapeutic efficacy. Key constraints include: high donor-dependent heterogeneity; rapid loss of stemness during *in vitro* expansion; restricted differentiation capacity; poor long-term survival in the harsh IVD microenvironment; and failure to enable structural reconstruction of the NP and functional regeneration of notochordal cells (Xiang et al., 2025). These limitations have driven the investigation of a broad array of alternative stem cell populations for DDD therapy, including pluripotent stem cells and tissue-specific adult stem cells. Relative to MSCs, these alternative cell sources present distinct advantages and limitations across four core dimensions: lineage specification, safety profile, clinical translatability, and regenerative capacity for IVD repair.

Embryonic stem cells (ESCs) and induced pluripotent stem cells (iPSCs) are the two primary classes of pluripotent stem cells. Unlike MSCs, which are restricted to mesodermal lineages, ESCs and iPSCs exhibit trilineage differentiation potential, giving rise to cell types of all three embryonic germ layers (Zhang et al., 2022). ESCs display *bona fide* pluripotency and unlimited self-renewal capacity. They undergo robust, reproducible differentiation into notochordal-like and NP-like cells, which recapitulate the molecular and functional phenotypes of juvenile native NP cells (Diaz‐Hernandez et al., 2020). This property enables structural regeneration of the intervertebral disc. Furthermore, ESCs can be expanded indefinitely *in vitro* with stable retention of karyotype and stemness (Amit et al., 2000). This eliminates the donor heterogeneity and progressive stemness attenuation that are inherent limitations of primary MSCs, and supports the production of standardized, clinical-grade cell products. However, the derivation of human ESCs requires the destruction of pre-implantation human embryos. This has raised intractable ethical concerns and prompted legal restrictions in many jurisdictions, which severely impede their clinical translation. Additional safety barriers remain. First, residual undifferentiated ESCs carry a substantial risk of *in vivo* teratoma formation (Blum and Benvenisty, 2008). Second, allogeneic ESCs induce robust immune rejection *via* major histocompatibility complex (MHC) mismatch, necessitating lifelong immunosuppressive treatment (Koch et al., 2008). Together, these ethical and safety limitations make ESCs a far less favorable candidate than MSCs for the clinical treatment of DDD.

Generated *via* somatic cell reprogramming, iPSCs combine the pluripotent differentiation potential of ESCs with the absence of ethical constraints, making them a promising alternative to MSCs. Like ESCs, iPSCs can be directionally differentiated into functional NP cells and notochordal cells (Sheyn et al., 2019). This enables structural repair of degenerative intervertebral discs, an outcome unachievable by MSCs through paracrine effects alone. Autologous iPSCs derived from patient somatic cells support personalized therapy and fully eliminate immune rejection (Madrid et al., 2021). This confers an unparalleled advantage over allogeneic MSCs and ESCs. Additionally, the reprogramming process resets cellular senescence. It restores telomere and mitochondrial function to generate youthful, functional cells that outperform MSCs from aged donors (Spitzhorn et al., 2019). iPSCs also exhibit high compatibility with CRISPR-Cas9 gene editing, enabling correction of pathogenic mutations to achieve synergistic regenerative and gene therapy (Ben Jehuda et al., 2018). However, iPSCs face critical barriers to clinical translation compared with mature, readily accessible MSCs. These barriers include teratoma formation from residual undifferentiated cells, genomic instability during reprogramming, lengthy production cycles, high manufacturing costs, and epigenetic memory that impairs differentiation stability (Fakoya et al., 2022).

Tissue-specific adult stem cells—including hematopoietic stem cells (HSCs), muscle stem cells (MuSCs), epithelial stem cells (EpSCs), and germline stem cells (GSCs)—exhibit high lineage fidelity and targeted regenerative capacity within their native tissues. However, their highly restricted differentiation potential renders them unsuitable for broad DDD therapy. These tissue-specific adult stem cells lack the three core attributes that make MSCs effective for DDD therapy: multipotent versatility, robust chondrogenic potential, and adaptability to the IVD microenvironment. Accordingly, they cannot serve as competitive alternatives to MSCs for clinical DDD therapy.

## 4 Molecular mechanisms of MSC-derived Exos in DDD

**FIGURE 3:**
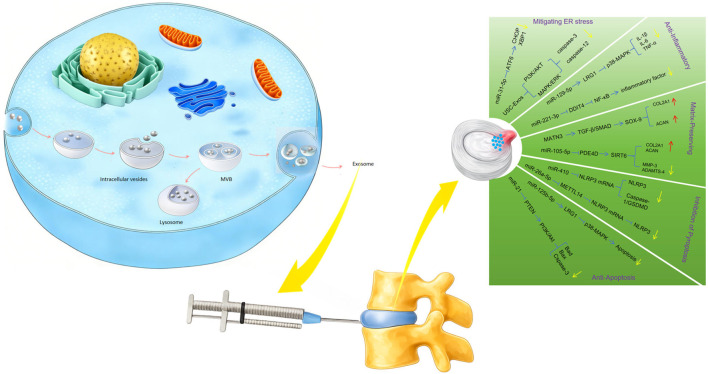
Depicts the process of exosome generation, their injection into the IVD, and the resulting therapeutic effects in disc degeneration.

MSCs also exhibit their therapeutic potential through paracrine signaling, releasing bioactive molecules that influence surrounding cells and tissues. Among these molecules, Exos are key mediators of intercellular communication (Krut et al., 2021). These nanoscale vesicles (30–150 nm) carry a complex cargo of proteins, lipids, and genetic material, including miRNAs, mRNAs, and DNA, facilitating sophisticated communication between cells (Wu et al., 2024). Exos derived from MSCs play a crucial role in tissue repair by modulating inflammation, supporting ECM production, and enhancing cell survival through various molecular mechanisms (Figure 3).

### 4.1 Exo-mediated regulation of endoplasmic reticulum stress in DDD

Degenerative stressors—such as ROS, inflammatory cytokines, abnormal mechanical load, and metabolic disturbances—trigger the accumulation of misfolded proteins, activating the unfolded protein response (UPR). Sustained UPR activation, particularly through the PERK-eIF2α-ATF4-CHOP axis, initiates intrinsic apoptotic signaling *via* the CHOP-caspase-12/3 axis, contributing to NP cell loss and ECM depletion. Recent studies show that Exos derived from various stem cells can effectively alleviate endoplasmic reticulum (ER) stress by delivering specific regulatory molecules, offering potent cytoprotective effects and preserving disc homeostasis.

Multiple studies indicate that Exos alleviate ER stress by modulating critical pro-survival signaling pathways. Liao et al. showed that BMSC-derived Exos attenuated AGE-induced ER stress in human NP cells and reduced the expression of key UPR markers (Liao et al., 2019). Their inhibitor experiments suggested that these protective effects were partly mediated by PI3K/AKT and ERK signaling, as blockade of either pathway diminished the Exos-induced suppression of CHOP and caspase activation. Likewise, Xiang et al. reported that human urine-derived MSC-Exos alleviated pressure-induced ER stress in NP cells, including reduced GRP78 and CHOP expression (Xiang et al., 2020). Pharmacological inhibition further supported the involvement of AKT and ERK signaling in this process. In a rat tail puncture model, intradiscal injection of urine-derived MSC (UMSC)-derived Exos effectively delayed DDD progression, as evidenced by preserved disc height, lower degenerative scores on MRI, and reduced expression of ER stress and apoptosis markers *in vivo* (Guo et al., 2021). These findings highlight UMSC-derived Exos, sourced non-invasively, as promising therapeutic agents capable of interrupting the ER stress-apoptosis nexus in DDD.

Beyond activating broad pro-survival kinases, the specific molecular cargo of Exos, particularly miRNAs, can directly target and silence core components of the UPR machinery. A prime example is the targeting of activating transcription factor 6 (ATF6), one of the major UPR sensors. Xie et al. demonstrated that MSC-derived Exos are enriched with miR-31-5p (Xie et al., 2020). Upon internalization by endplate chondrocytes under oxidative stress, miR-31-5p directly binds to the 3′ UTR of ATF6 mRNA, inhibiting its expression. Downregulation of ATF6 disrupts the transcriptional activation of downstream ER stress effectors, such as CHOP, XBP1, and GRP78, inhibiting ER stress-induced apoptosis and calcification. This mechanism highlights the precision with which exosomal miRNAs can modulate specific branches of the UPR.

Collectively, these studies show that Exos from MSCs alleviate pathological ER stress in disc cells through a multi-tiered strategy: convergent activation of pro-survival PI3K/AKT and MAPK/ERK signaling pathways to inhibit apoptosis; silencing of specific UPR components to downregulate the CHOP-mediated terminal apoptotic cascade; and enhanced resilience to stressors that trigger proteostatic imbalance. By maintaining ER homeostasis and enhancing NP cell viability, Exo-based interventions target a fundamental driver of disc cell death, providing a potent and versatile strategy to decelerate DDD progression and preserve disc integrity.

### 4.2 MSC-Exos targeting apoptosis in DDD

A key therapeutic action of MSC-Exos in DDD is their ability to mitigate NP cell apoptosis. This anti-apoptotic effect is achieved through the precise delivery of regulatory molecules, including microRNAs (miRNAs), proteins, and lipids, which modulate pro-survival signaling pathways and inhibit apoptotic executors, thereby restoring cellular homeostasis in the degenerative disc environment.

The bioactive cargo of MSC-Exos, especially miRNAs, plays a pivotal role in modulating intrinsic and extrinsic apoptotic cascades. Cheng et al. demonstrated that MSC-Exos are enriched with miR-21, which is efficiently transferred to NP cells under TNF-α stimulation (Cheng et al., 2018). Mechanistically, miR-21 promotes cell survival by targeting phosphatase and tensin homolog (PTEN), a key negative regulator of cellular viability. PTEN inhibition activates the PI3K/Akt signaling axis, a central mediator of cell survival. This activation leads to phosphorylation and inactivation of the pro-apoptotic protein Bad, while repressing downstream apoptotic effectors such as Bax and cleaved caspase-3, thereby preventing TNF-α-induced NP cell apoptosis. In a complementary mechanism, Duan et al. showed that MSC-Exos shuttle miR-125b-5p, which binds the 3′ untranslated region (3′UTR) of TNF receptor-associated factor 6 (TRAF6) mRNA (Duan et al., 2023). Downregulation of TRAF6, a key adapter protein in the NF-κB pathway, suppresses pro-inflammatory and pro-apoptotic signaling, shifting the balance of Bcl-2 family proteins toward survival. This is evidenced by reduced Bax and cleaved caspase-3 levels, and increased Bcl-2 expression. Knockdown of miR-125b-5p in MSC-Exos abolished this anti-apoptotic effect, confirming its essential role. Additionally, Cui and Zhang identified miR-129-5p as another critical effector in MSC-derived Exos (Cui and Zhang, 2021). By targeting the 3′UTR of leucine-rich α-2-glycoprotein 1 (LRG1) mRNA, miR-129-5p represses LRG1 expression, inhibiting the pro-apoptotic p38 MAPK pathway and reducing NP cell death in an IL-1β-induced inflammatory model.

Other exosomal miRNAs contribute to anti-apoptotic effects. Zhu et al. showed that BMSC-Exos deliver miR-532-5p, which targets RASSF5 to inhibit apoptosis and ECM degradation (Zhu G. et al., 2020). Shi et al. reported that BMSC-Exos increase miR-155 expression, which downregulates Bach1, upregulating HO-1, and activating protective autophagy, thus inhibiting apoptosis under low nutrient supply (Shi et al., 2021). Additionally, Yuan et al. demonstrated that Exos from human placental MSCs, combined with an antagonist of miR-4450, enhance protective effects by upregulating ZNF121, further reducing NP cell apoptosis (Yuan et al., 2020).

The anti-apoptotic efficacy of MSC-Exos is validated against the multiple stressors in the degenerated disc microenvironment, including ER stress, oxidative stress, and inflammatory cytokines. Liao et al. showed that MSC-Exos protect NP cells from AGE-induced apoptosis driven by ER stress (Liao et al., 2019). Similarly, Xia et al. demonstrated that BMSC-Exos alleviate oxidative stress by reducing ROS accumulation and restoring mitochondrial membrane potential, counteracting H_2_O_2_-induced mitochondrial apoptosis (Xia et al., 2022). Hu et al. further confirmed that BMSC-Exos inhibit compression-induced NP cell apoptosis by mitigating oxidative damage, preserving mitochondrial integrity under biomechanical stress (Hu et al., 2021).

In summary, MSC-Exos employ a multi-pronged strategy to combat NP cell apoptosis. They deliver anti-apoptotic miRNAs (e.g., miR-21, miR-125b-5p, miR-129-5p, miR-532-5p) that target critical apoptotic pathways (PTEN/PI3K/Akt, TRAF6/NF-κB, LRG1/p38 MAPK). Concurrently, they activate pro-survival signaling (AKT, ERK) to counteract apoptotic inducers such as ER stress, oxidative damage, inflammatory cytokines, and biomechanical stress. By intervening at multiple levels of the apoptotic machinery, MSC-Exos enhance NP cell resilience, suppress caspase activation, and promote a regenerative niche, positioning them as a potent, cell-free therapeutic modality for DDD.

### 4.3 Exosomal suppression of pyroptosis in DDD

Accumulating evidence highlights the pivotal role of MSC-Exos in suppressing pyroptosis, an intensely inflammatory form of programmed cell death, within NP cells, thereby attenuating DDD progression. The NLRP3 inflammasome is a central mediator in this pathological cascade, and Exos deploy a range of molecular mechanisms to target its activation, including gene silencing, upstream signaling modulation, and epigenetic regulation. Zhang et al. provided compelling evidence that NLRP3-mediated pyroptosis is aberrantly activated in both *in vivo* and *in vitro* DDD models. Their findings showed that MSC-Exos efficiently deliver miR-410 to NP cells, where it directly binds the 3′ untranslated region (3′UTR) of NLRP3 mRNA (Zhang et al., 2020). This binding suppresses NLRP3 inflammasome assembly, reducing caspase-1 cleavage and its downstream effector GSDMD. Consequently, the maturation and secretion of pro-inflammatory cytokines IL-1β and IL-18 are attenuated, alleviating disc degenerative changes. Similarly, Yuan et al. found that exosomal miR-26a-5p from hUCMSCs mitigates DDD by targeting methyltransferase-like 14 (METTL14), which reduces the N^6^-methyladenosine (m^6^A)-mediated stabilization of NLRP3 mRNA, suppressing its translational expression. This pathway inhibits NP cell pyroptosis, as evidenced by reduced cleaved caspase-1, GSDMD fragments, and IL-1β/IL-18 levels. These findings reveal a novel RNA epigenetic regulatory axis with significant implications for disc degeneration therapy (Yuan et al., 2021).

Beyond direct mRNA targeting, Exos regulate NLRP3 through upstream signaling and epigenetic mechanisms. Xia et al. showed that BMSC-Exos inhibit ROS production, a key activator of the NLRP3 inflammasome, reducing NP cell apoptosis and preventing inflammasome priming (Xia et al., 2022). Yuan et al. identified a novel epigenetic pathway in which exosomal miR-26a-5p from UCMSCs targets METTL14, a key component of the m6A methyltransferase complex. Downregulation of METTL14 impairs NLRP3 mRNA stability and translation through an IGF2BP2-dependent mechanism, leading to decreased NLRP3 protein expression and subsequent inhibition of NP cell pyroptosis (Yuan et al., 2021). Zhao et al. showed that Exos from MSCs preconditioned in hypoxic and inflammatory environments are enriched with miR-221-3p, which targets DDIT4 to suppress the NF-κB pathway (Zhao et al., 2025). Since NF-κB signaling is essential for NLRP3 inflammasome priming, this represents another potent mechanism for Exo-mediated pyroptosis suppression.

In addition to counteracting pyroptosis, MSC-Exos protect against other forms of programmed cell death linked to DDD. Chen et al. demonstrated that BMSC-Exos inhibit ferroptosis in NP cells induced by tert-butyl hydroperoxide (TBHP) or RSL3. This protection occurs through activation of the p62-KEAP1-NRF2 antioxidant pathway, which upregulates key anti-ferroptotic proteins, including glutathione peroxidase 4 (GPX4) and ferritin heavy chain 1 (FTH1), and downregulates pro-ferroptotic markers such as ACSL4 and PTGS2 (Chen C. et al., 2025). The efficacy of this mechanism was further validated *in vivo*, where Exo treatment preserved disc structure and reduced ferroptosis markers in a puncture-induced rat DDD model, with effects comparable to the ferroptosis inhibitor Ferrostatin-1 (Fer-1).

### 4.4 Exosomal modulation of inflammation in DDD

Exos serve as central regulators in remodeling the IVD immune microenvironment, orchestrating the fine-tuning of key inflammatory pathways and immune cell polarization. This modulation drives a transition from a catabolic, pro-inflammatory milieu to an anabolic, anti-inflammatory, pro-regenerative state—critical for arresting or reversing DDD progression. A core mechanism of Exos’ anti-inflammatory efficacy is their precise modulation of inflammasome activation and downstream signaling, targeting the inflammatory response (Xia et al., 2022).

Exosomal miRNAs play a pivotal role in directly interfering with these pathways. For instance, Yuan et al. demonstrated that Exos derived from hUCMSCs, enriched with miR-26a-5p, inhibit the N6-methyladenosine (m6A) “writer” protein METTL14 (Yuan et al., 2021). This inhibition reduces m6A methylation and stabilizes NLRP3 mRNA, suppressing inflammasome assembly, caspase-1 activation, and the maturation of pro-inflammatory cytokines IL-1β and IL-18, thus mitigating NP cell pyroptosis and ECM degradation. Similarly, BMSC-Exos deliver miR-129-5p, targeting Leucine-Rich Alpha-2-Glycoprotein 1 (LRG1), a key regulator of the p38 MAPK pathway. Inhibiting this pathway downregulates pro-inflammatory cytokines (e.g., IL-1β, IL-6, TNF-α) in degenerated NP cells (Cui and Zhang, 2021). Exosomal miRNAs also modulate other critical inflammatory hubs. MSC-derived miR-125b-5p targets TRAF6, inhibiting the NF-κB pathway and reducing the inflammatory response (Duan et al., 2023). Additionally, Exos from MSCs preconditioned in hypoxic and inflammatory environments are enriched with miR-221-3p, which epigenetically silences DDIT4, suppressing NF-κB signaling and alleviating NP cell senescence (Zhao et al., 2025).

In DDD, the pathological immune landscape is dominated by pro-inflammatory M1 macrophages, which exacerbate tissue breakdown through the release of catabolic enzymes and cytokines. Exos have emerged as potent mediators of immune reprogramming. MSC-derived Exos deliver miR-129-5p to alleviate IDD *via* targeting LRG1 and inhibiting p38 MAPK signaling, thus reducing NP cell apoptosis, ECM degradation, and macrophage M1 polarization (Cui and Zhang, 2021). This axis mitigates NP cell apoptosis and ECM degradation, while promoting M1-to-M2 macrophage polarization, as evidenced by decreased M1 markers (CD86, iNOS) and increased M2 markers (CD206, ARG1). However, Exos from degenerated NP cells may have opposing effects. For example, they may deliver miR-27a-3p, which suppresses the PPARγ/NF-κB/PI3K/AKT pathway in macrophages, promoting M1 polarization and exacerbating inflammation-driven degeneration (Zhao et al., 2023).

The anti-inflammatory power of MSC-Exos lies in their diverse cargo, including specific miRNAs (e.g., miR-146, miR-155, miR-181) and immunomodulatory proteins (e.g., TSG-6, CD73) (Chen et al., 2024a; Bhujel et al., 2022; Bhat et al., 2025). These molecules work together to downregulate pro-inflammatory cytokines and promote the secretion of anti-inflammatory mediators like IL-10 and TGF-β. Chen et al. showed that MSC-Exos suppress H_2_O_2_-induced NLRP3 inflammasome activation and reduce IL-1β and GSDMD levels in NP cells (Yu et al., 2023). Similarly, MSC-Exos and their secretome significantly reduce IL-1α, IL-1β, IL-6, IL-17, and TNF-α secretion in degenerative NP and AF cells, attributed to the inhibition of NF-κB nuclear translocation (Zhao et al., 2025; Liang et al., 2022). Notably, conditioned medium from ADMSCs—which contains Exos, soluble proteins, and growth factors—exerts a more potent anti-inflammatory and anti-catabolic effect than isolated Exos or the soluble fraction alone (González-Cubero et al., 2022). This synergy creates a regenerative microenvironment conducive to tissue repair.

### 4.5 Matrix preservation by MSC-Exos in DDD

MSC-Exos drive the preservation and restoration of ECM homeostasis in the IVD—an essential process for halting or reversing disc degeneration. Their therapeutic efficacy is achieved through a dual-pronged strategy that enhances matrix anabolism while suppressing catabolism, thereby rectifying the imbalance central to DDD pathology. On the anabolic side, MSC-Exos stimulate NP cell biosynthesis, reprogramming them toward a matrix-producing, chondrogenic progenitor-like state. Consistent evidence shows that Exo treatment upregulates key anabolic genes and proteins, including ACAN, collagen type II, and the chondrogenic transcription factor SOX-9 (Bhujel et al., 2022; Lu et al., 2017). For example, Zhang et al. demonstrated that ADMSC-Exos promoted the expression of these chondrogenic markers in human NP cells over 21 days of culture (Zhang Z. et al., 2021). Similarly, Chen et al. showed that BMSC-Exos increased anabolic markers and correlated with enhanced proteoglycan deposition (Xia et al., 2019). This pro-anabolic reprogramming can also reverse cellular dysfunction linked to aging and degeneration. For instance, Sun et al. found that small Exos from iPSC-derived MSCs transfer miR-105-5p into senescent NP cells, targeting and downregulating PDE4D. This activates the Sirtuin 6 (SIRT6) pathway, which restores matrix synthesis (COL2A1, ACAN) and reduces degradation (MMP-3, ADAMTS-4), alleviating the degenerative phenotype (Sun et al., 2021). Additionally, Tilotta et al. reported that Exo-containing secretomes from both bone marrow and adipose-derived MSCs enhanced GAG synthesis in human NP cells under pro-inflammatory IL-1β stimulation, highlighting the robustness of this anabolic effect (Tilotta et al., 2023).

On the catabolic side, MSC-Exos inhibit ECM degradation by targeting matrix-degrading enzymes. They downregulate MMPs, such as MMP-1, MMP-3, and MMP-13. Chen et al. demonstrated that ASC-derived Exos slowed DDD progression by significantly reducing MMP-13 expression in the ECM (Chen D. et al., 2023). This inhibition is often achieved through specific miRNA delivery. For instance, exosomal miR-142-3p alleviates IL-1β-induced inflammation and catabolism in NP cells by targeting Mixed-Lineage Kinase 3 (MLK3), inhibiting the downstream MAPK pathway that drives MMP and ADAMTS expression (Zhu L. et al., 2020). Similarly, Zheng et al. showed that NP-derived Exos transferred miR-15a to downregulate MMP-3 *via* the PI3K/AKT and Wnt/β-catenin pathways (Zhang Q. et al., 2021). This catabolic inhibition is strategically paired with the modulation of endogenous inhibitors, such as TIMPs, to rebalance the catabolic/anabolic equilibrium in the degenerated disc microenvironment.

The matrix-preserving cargo of MSC-Exos is highly multifaceted. In addition to miRNAs, Exos are enriched with bioactive proteins and signaling molecules that directly stimulate ECM synthesis. A notable example is Matrilin 3 (MATN3), a cartilage-specific matrix protein. Guo et al. showed that UMSC-Exos, rich in MATN3, promoted NP cell proliferation and ECM synthesis (Guo et al., 2021). MATN3 acted as an extracellular trigger, activating the TGF-β/SMAD signaling pathway, which drives COL2A1 and ACAN expression. This highlights a direct, protein-mediated mechanism for anabolic stimulation independent of nucleic acid cargo. By delivering both anabolic stimulators and potent catabolic inhibitors, MSC-Exos comprehensively reprogram NP cells. This dual-action strategy restores ECM synthesis/degradation homeostasis, creating a favorable biochemical and molecular environment essential for the structural integrity and functional restoration of the degenerated IVD.

In summary, MSC-Exos have shown significant therapeutic potential for DDD by targeting various pathological processes, including apoptosis, senescence, ER stress, inflammation, ECM degradation, pyroptosis, oxidative stress, and impaired chondrogenesis. Their cell-free nature, low immunogenicity, and potential for enhancement *via* preconditioning, engineering, or biomaterial-assisted delivery make them promising next-generation therapeutic candidates (Liang et al., 2022; Hu et al., 2022). However, challenges related to standardized isolation, quantification, dosing, and targeted delivery must be addressed for successful clinical translation.

## 5 Functional enhancement of therapeutic agents: engineering strategies

### 5.1 Hydrogels

**FIGURE 4:**
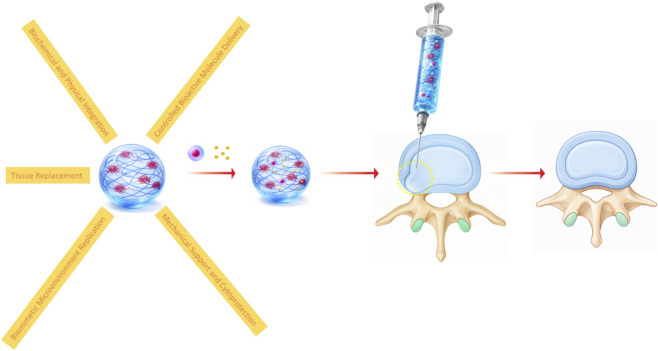
Depicts the mechanism by which hydrogels, in combination with MSCs, are utilized to treat DDD.

Hydrogels are pivotal in advancing MSC-based therapies for DDD, acting as multifunctional platforms that enhance cell retention, viability, and directed differentiation within the challenging disc microenvironment, while also modulating pathological conditions and providing essential mechanical support. Their high water content, tunable viscoelasticity, and ability to incorporate bioactive cues make them ideal for mimicking native NP tissue and counteracting hypoxia, acidity, oxidative stress, nutrient deficiency, and elevated lactate levels—key hallmarks of the degenerative disc niche (Figure 4).

As a “cellular sanctuary,” hydrogels significantly boost MSC survival, engraftment, and retention through physical shielding and biochemical modulation. In rat IDD models, RGD peptide-modified polysaccharide hydrogels act as a 3D “cell shelter” that buffers dynamic mechanical loads, retaining intense fluorescent signals from encapsulated NPMSCs for 30 days post-implantation, in contrast to rapid signal loss in the cell-only group, demonstrating prolonged MSC retention in the hostile degenerative disc (Wang et al., 2019). Thermosensitive hydrogels further protect MSCs by undergoing sol–gel transition at body temperature, encapsulating cells to shield them from shear forces during injection and forming a physical barrier against pro-inflammatory cytokines (Wang et al., 2019). Hydrogels also actively mitigate microenvironmental stressors to enhance cell viability: hypoxia-inducible interpenetrating polymer network (IPN) hydrogel microspheres create and maintain a localized hypoxic niche for up to 5 days *via* laccase-mediated oxygen consumption, activating the HIF-1α signaling pathway to boost the survival and functional activity of both exogenous MSCs and endogenous stem cells (Zhou et al., 2025). Antioxidant peptides loaded in gelatin-fibrin-alginate hydrogel microspheres scavenge ROS and activate the NRF2/HO-1 antioxidant pathway, directly alleviating oxidative stress to promote BMSC survival (Chen Y. et al., 2025). Lactate oxidase–MnO_2_ nanozyme-loaded NP matrix hydrogel microspheres (LMGDNPs) catalytically convert accumulated lactate into pyruvate while decomposing H_2_O_2_, relieving both acidosis and oxidative stress; this catalytic cycle forms a self-sustaining benign loop, nearly doubling BMSC viability under simulated degenerative conditions and facilitating synergistic repair of endogenous and exogenous cells (Peng et al., 2024).

Beyond cytoprotection, hydrogels act as “directors” of MSC fate, guiding their differentiation toward an NP-like phenotype through intrinsic physical properties, bioactive factor delivery, and matrix-derived biological signals. Sustained, local release of growth factors is a core strategy for directed differentiation: PLGA microspheres loaded with GDF5 and incorporated into dECM/chitosan hybrid hydrogels enable slow GDF5 release over 2–3 weeks, significantly upregulating SOX9, COL2A1, and ACAN expression in rat NPMSCs, promoting chondrogenic differentiation, NP-like tissue formation, and effective disc repair both *in vitro* and *in vivo* (Ma et al., 2024). Peptide-functionalized double-network hydrogels also drive NP-like differentiation *via* sustained GDF5 release, as evidenced by increased SOX9 and KRT19 expression and enhanced proteoglycan and collagen deposition (Ho et al., 2023). Hydrogels loaded with TGF-β3 similarly upregulate NP-specific markers in MSCs and stimulate proteoglycan-rich ECM synthesis (Ma et al., 2025).

DECM hydrogels, rich in native collagens, glycosaminoglycans, and matrix-bound bioactive cues, serve as a natural “blueprint” for regeneration. dECM/chitosan hybrid hydrogels preserve essential NP matrix components and exhibit mechanical properties matching healthy NP tissue, supporting MSC adhesion, proliferation, and tissue-specific differentiation without exogenous growth factors (Chen et al., 2024a). dECM hydrogel microspheres derived from NP tissue further enhance MSC viability and NP-specific differentiation, highlighting the critical role of matrix-derived signals in guiding stem cell fate (Peng et al., 2024). Thermosensitive hydrogels composed of porcine NP dECM loaded with ADSC Exos maintain NP cell viability, inhibit apoptosis, and regulate ECM metabolism by reducing MMP activity, offering a clinically translatable strategy for disc preservation (Xing et al., 2021).

The intrinsic physical and mechanical properties of hydrogels are equally critical for directing MSC behavior. Photo-crosslinkable hydrogels (e.g., gelatin methacryloyl (GelMA), hyaluronic acid methacrylate (HAMA)) allow precise control over mechanical stiffness and network architecture, facilitating nutrient diffusion and optimizing cell–matrix interactions essential for MSC metabolic activity and ECM deposition (Chen Y. et al., 2025). Alginate hydrogels with fast stress-relaxation properties—mimicking the viscoelasticity of healthy native NP—induce BMSC differentiation into NP-like cells without exogenous factors *via* the integrin-FAK-ROCK signaling axis, significantly upregulating KRT19 and FOXF1 while suppressing osteogenic/adipogenic differentiation. Viscoelastic hydrogels with stress-relaxing capabilities also replicate the dynamic mechanical environment of healthy NP, activating mechanosensitive ion channels and downstream pathways that drive NP-like differentiation (Liu et al., 2024). Functionalization with bioactive sequences further refines differentiation and stress resistance: Sa12b-functionalized self-assembling peptide hydrogels alleviate acid-induced damage to NPMSCs by inhibiting acid-sensing ion channels and the downstream Ca^2+^/p-ERK pathway, enhancing MSC bioactivity under low-pH conditions (Han et al., 2022); injectable self-assembling peptide hydrogels modified with Link N or BMP-7 mimetic peptides direct MSC differentiation toward an NP phenotype while supporting long-term ECM remodeling (Zhang et al., 2025a). RGD peptide modification universally improves MSC adhesion and spreading *via* integrin binding, a prerequisite for cell survival, mechanical signal transduction, and sustained function (Chen et al., 2024b).

Importantly, emerging long-term *in vivo* evidence suggests that some hydrogel-assisted disc repair strategies are well tolerated, but safety concerns have not been fully resolved. In a 12-month ovine model, ADMSC-loaded chitosan/carboxymethyl cellulose hydrogel stabilized disc height and mitigated degeneration without overt neurological deficits or abnormal behavior, indicating acceptable long-term tolerance in a large-animal setting (Schmitt et al., 2021). Similarly, ADMSC-loaded collagen hydrogel maintained disc height at 6 and 12 months, although full structural restoration was not achieved (Friedmann et al., 2021). Selected biomaterials also appear to have favorable biological safety profiles. In a sheep discectomy model, an ultra-purified alginate bioresorbable gel showed low endotoxicity, no evident immunogenicity, no material protrusion, and no neoplastic changes during follow-up, supporting the importance of material purity and biomechanical compatibility (Ukeba et al., 2022). However, hydrogel-related risks remain clinically relevant. In a prospective study of an injectable hydrogel implant for lumbar DDD, partial implant migration occurred in 5 of 83 treated discs, requiring endoscopic removal (Clerk-Lamalice et al., 2025). In addition, PRP hydrogel used after PELD showed no severe adverse events during follow-up, but potential hydrogel outflow through annular fissures remained difficult to quantify (Zhang et al., 2023). These findings indicate that implant containment, annular integrity, and long-term mechanical matching remain critical for translation.

Collectively, hydrogels enhance MSC-mediated disc repair through the synergistic integration of biomimetic design, microenvironmental conditioning, bioactive functionalization, and mechanical support. By improving cell retention, mitigating degenerative stressors, guiding lineage-specific differentiation, and restoring matrix homeostasis and biomechanical function, hydrogel–MSC constructs have demonstrated consistent efficacy in preclinical models of DDD. Overall, current long-term studies support the potential safety of hydrogel-based MSC delivery, but systematic evaluation of disc biomechanics, inflammatory responses, implant migration, and degradation-related effects is still limited. Future translational studies should therefore incorporate these long-term safety endpoints alongside regenerative efficacy. Future development should prioritize the design of smart hydrogels with responsive degradation kinetics, patient-specific mechanical tailoring, and integrated multi-modal regulation (e.g., combined growth factor delivery, antioxidant activity, and mechanical sensing) to address the complexity of the degenerative disc niche and accelerate the clinical translation of MSC-based hydrogel therapies for DDD.

### 5.2 Genetic modification

Genetic modification is expanding the therapeutic scope of MSCs and their derived Exos in DDD. Although MSCs have clear regenerative potential, their efficacy in the degenerative IVD is constrained by limited persistence and functional instability. Gene-engineering strategies are therefore being used to enhance lineage specification, matrix regulation, stress tolerance, immunomodulation and exosomal function. CRISPR-based platforms further extend this framework by enabling more precise and potentially controllable genetic modulation. Together, these advances position engineered MSCs and MSC-derived Exos as a more versatile therapeutic platform, although translation remains limited by delivery, manufacturing and safety constraints (Table 1).

**TABLE 1:** Representative genetic modification strategies.

Therapeutic focus	Gene target(s)	Engineering method	Representative model	Expected/Observed therapeutic outcomes	References
NP-like lineage specification	SOX9 + SIX1	Plasmid overexpression/electroporation in hUC-MSCs	*In vitro* chondrogenic induction and rat IVDD transplantation	Enhanced NP-like differentiation and disc repair.	Khalid et al. (2022)
Matrix synthesis and stable chondrogenic programming	ZNF865	CRISPRa-mediated targeted gene activation in hASCs (with matrix-directed CRISPRa background)	Pellet culture and tissue-engineered IVD constructs	Increased matrix deposition and improved mechanical maturation without hypertrophy.	Levis et al. (2025)
Growth factor-driven matrix regeneration	TGF-β1, IGF-1, BMP7	CRISPR/Cas9-mediated safe-harbor knock-in with Tet-off expression system	Tonsil-derived MSCs in rat IVDD	Promoted NP regeneration, matrix restoration, and reduced inflammation/pain.	Kim et al. (2023)
Receptor/signaling optimization for NP-like differentiation	BMPR2/GDF6 axis	BMPR2 upregulation or BMPR2-high cell selection with GDF6 stimulation	Human adipose-derived stem cells in 3D culture	Strengthened NP-like differentiation and proteoglycan-rich matrix formation.	Hodgkinson et al. (2020)
Cell-free cytoprotection under inflammatory stress	HO-1	HO-1 overexpression in donor BMSCs to generate engineered Exos	IL-1β-stimulated NP cells	Reduced NP cell apoptosis, senescence, and inflammatory signaling.	Zhang et al. (2025c)
Oxidative stress resistance in MSCs	Sod2; Cat	Adenoviral transduction in hADSCs	Mouse needle-puncture IVDD	Improved oxidative stress resistance, matrix preservation, and inflammation control.	Xiao et al. (2021)
Immune evasion and enhanced persistence	B2M	CRISPR-Cas9 knockout, preferably *via* RNP delivery	Human umbilical cord MSCs under allogeneic inflammatory conditions	Reduced immune recognition and improved MSC survival and immunomodulation.	Han et al. (2024)
Anti-inflammatory Exo engineering	TSG-6	CRISPRa-SAM lentiviral activation of TSG-6 in MSCs	IL-1β-stimulated human IVD cells *in vitro*	Enhanced anti-inflammatory EV activity and reduced pro-inflammatory gene expression	Martinez-Zalbidea et al. (2025)

Genetic modification in MSC-based therapy for DDD is primarily intended to promote NP-like lineage specification and re-establish matrix homeostasis within the degenerative IVD microenvironment. The former has largely focused on regulators of chondrogenesis and NP development. Among these, SOX9 represents the most extensively characterized target. In hUCMSCs, SOX9 overexpression enhanced anabolic factor expression and promoted chondroprogenitor-like differentiation, whereas co-expression of SOX9 and SIX1 further reinforced NP-like lineage commitment and improved disc repair *in vivo* (Khalid et al., 2022). Additional targets have also been investigated to enhance matrix synthesis while constraining hypertrophic drift, which remains a major obstacle to stable chondrogenic programming. In this regard, ZNF865 promoted ADMSC proliferation and increased GAG and collagen II production in tissue-engineered IVD constructs without inducing hypertrophic markers, including COL10A1, RUNX2 and MMP13 (Levis et al., 2025). These effects were further associated with improved mechanical properties, as evidenced by reduced compressive strain and increased stiffness.

Enhance matrix anabolism mainly through growth factor–mediated genetic engineering. Within this framework, members of the TGF-β superfamily have attracted particular interest owing to their established roles in ECM synthesis and NP phenotypic regulation. Consistent with this, tonsil-derived MSCs engineered to co-express TGF-β1, IGF-1 and BMP7 exhibited superior therapeutic efficacy compared with unmodified cells or single-factor engineered counterparts in a rat IVDD model. These triple-modified MSCs promoted NP regeneration, increased ACAN and COL2A1 expression, suppressed inflammatory and nociception-related mediators, and maintained cell viability together with transgene expression over 6 weeks (Kim et al., 2023). GDF6 has likewise emerged as a promising target for promoting NP-like differentiation. Human adipose-derived MSCs exhibited higher and more stable BMPR2 expression than donor-matched bone marrow MSCs, together with stronger GDF6-induced activation of SMAD1/5/8 and ERK1/2 signalling. BMPR2 upregulation further promoted NP-related marker expression and proteoglycan-rich matrix deposition. Mechanistically, SMAD1/5/8 signalling was required for NP-like differentiation, whereas ERK1/2 signalling mainly supported anabolic gene expression and matrix production (Hodgkinson et al., 2020). Collectively, these findings indicate that genetic engineering can enhance MSC-mediated disc regeneration through coordinated regulation of lineage fidelity and matrix anabolism, although the available evidence remains predominantly preclinical.

Genetic modification has also been explored as a means of improving MSC persistence and functional stability within the hostile disc microenvironment. In this setting, engineered MSCs may not only better tolerate degenerative stress but also exert indirect protective effects on resident NP cells. One example is HO-1, a cytoprotective enzyme with a central role in antioxidant defence. MSC-derived Exos from HO-1-overexpressing BMSCs were shown to reduce apoptosis, senescence and cell-cycle arrest in IL-1β-stimulated NP cells, while also suppressing NF-κB activation (Zhang H. et al., 2025). Similarly, MSCs overexpressing Sod2 or Cat, which encode key enzymes involved in reactive oxygen species clearance, showed increased proliferative capacity and higher expression of NP-associated markers, including SOX9, ACAN and COL2A1, compared with unmodified controls (Xiao et al., 2021). In a mouse needle-puncture IVDD model, transplantation of these modified MSCs was associated with better preservation of disc height, higher MRI T2 signal intensity and improved histological features, together with increased GAG and COL2A1 deposition and reduced expression of inflammatory mediators such as IL-1β, IL-6 and TNF-α (Kim et al., 2023). These studies support antioxidant-oriented genetic modification as a means of improving MSC survival and reparative activity in the degenerative IVD, although the evidence remains largely preclinical.

Chronic low-grade inflammation is another central driver of DDD progression and has therefore become a target for engineering MSC immunomodulatory function. One challenge in allogeneic MSC therapy is that inflammatory cues such as IFN-γ can increase MHC class I expression and thereby promote CD8^+^ T cell-mediated clearance. In this context, disruption of B2M, an essential component of MHC class I, has emerged as a strategy to reduce immune recognition. In human umbilical cord MSCs, CRISPR–Cas9-mediated B2M disruption eliminated MHC class I surface expression, suppressed CD8^+^ T cell proliferation, and improved cell survival under allogeneic inflammatory conditions. The edited cells also exhibited enhanced expression of immunoregulatory mediators after stimulation, suggesting the concurrent promotion of immune evasion and anti-inflammatory capacity (Han et al., 2024). Additionally, CRISPR-dCas9 activation of TSG-6 in MSCs can effectively remodel the cargo of secreted extracellular vesicles, significantly enhancing their anti-inflammatory effects on human degenerative intervertebral disc cells and providing a new strategy to improve the therapeutic efficacy of MSC-derived vesicles for DDD (Martinez-Zalbidea et al., 2025).

Although genetically modified MSCs have shown therapeutic promise in DDD, their translation requires careful control of three major safety issues: off-target effects, long-term genetic stability and tumorigenic risk (Baum et al., 2011). Current preclinical evidence suggests that these concerns can be mitigated through vector choice, editing-platform design and stepwise safety validation (Vakulskas et al., 2018). For transgene-based approaches, the main risks are insertional mutagenesis and uncontrolled long-term expression, particularly in integrating lentiviral systems. Accordingly, several DDD studies have instead used non-integrating adenoviral vectors to achieve transient expression without genomic insertion (Zhang H. et al., 2025; Xiao et al., 2021). In parallel, lentiviral strategies have generally been accompanied by insertion-site assessment and serial passaging, with available studies reporting stable NP-like differentiation and no abnormal proliferation, ectopic tissue formation or malignant transformation *in vivo* (Hodgkinson et al., 2020; Khalid et al., 2022). For CRISPR-based editing, off-target activity remains the principal concern, but this can be reduced by transient RNP delivery, optimized sgRNA design and the use of dCas9-based CRISPRa/CRISPRi systems, which avoid double-strand DNA breaks and enable reversible transcriptional modulation (Bendixen et al., 2023).

Long-term stability and tumorigenicity have also been assessed in modified MSCs used for disc regeneration. Permanently edited MSCs have shown stable target modification during serial culture without evidence of chromosomal instability or immortalization, whereas dCas9-mediated regulation appears transient and less likely to induce persistent dysregulation of endogenous gene networks (Levis et al., 2025; Han et al., 2024). Reassuringly, multiple preclinical DDD studies have reported no uncontrolled proliferation, hypertrophic drift, ectopic calcification or tumor formation following transplantation of genetically modified MSCs or their derivatives (Khalid et al., 2022; Levis et al., 2025; Xiao et al., 2021). These findings support the overall preclinical safety of gene-modified MSC strategies, but the available evidence remains largely limited to short-to-term small-animal studies. Longer-term evaluation in large-animal models, together with standardized clinical-grade biosafety assessment, will therefore be essential before such approaches can be advanced toward human application.

Taken together, current evidence indicates that genetic modification can broaden the therapeutic scope of MSCs and MSC-derived Exos in DDD by targeting multiple aspects of disc degeneration, including lineage specification, matrix regulation, stress adaptation and inflammatory control. Preclinical studies suggest that these strategies can improve repair-related outcomes, and engineered Exos may provide a complementary cell-free means of delivering some of these effects. However, translation remains constrained by unresolved safety and manufacturing challenges. Future progress will depend on safer and more controllable delivery strategies, standardized manufacturing and quality-control frameworks, and more rigorous validation of long-term safety and efficacy in clinically relevant models. Greater attention to disease stage, biological heterogeneity and target selection will also be important for the rational development of genetically modified MSC- and Exo-based therapies for DDD.

### 5.3 Preconditioning

Preconditioning techniques, such as hypoxic culture conditions, are designed to enhance MSC survival and adaptability in challenging environments like the hypoxic IVD. These methods leverage MSCs’ ability to adapt to low oxygen, a critical trait for successful transplantation. Hypoxic preconditioning upregulates survival pathways, including stabilization of hypoxia-inducible factors, supporting cell viability under stress (Najar et al., 2019; Ferro et al., 2019). For example, hypoxic preconditioning with cobalt chloride upregulates HIF-1α and CXCR4, enhancing BMSC migration and homing to degenerated discs (Wang et al., 2018). Beyond survival, hypoxic preconditioning promotes MSC secretion of critical growth factors, such as VEGF, essential for tissue regeneration and vascularization (Fuentes et al., 2022). Studies show that MSCs preconditioned under hypoxia maintain their regenerative potential, improving integration and functionality in the nutrient-limited disc environment (Moeinabadi-Bidgoli et al., 2023). Hypoxic preconditioning (1%–3% O_2_) also promotes NP-like differentiation and improves cell survival in the disc’s hypoxic environment (Sinkemani et al., 2020). Moreover, preconditioning reduces apoptosis by modulating caspase-3 and Bcl-2 pathways, further enhancing transplanted cell retention (Wang et al., 2018).

In addition to hypoxia, other preconditioning strategies have been explored. Inducing quiescence through serum starvation enhances NP stem cell tolerance to nutrient deprivation, improving survival and matrix synthesis in degenerated discs (Chen Q. et al., 2023). Mechanical stimulation *via* swelling-mediated stretch using injectable UCST microgels upregulates mechanosensitive channels like TRPV4 and Piezo1, promoting NP-like differentiation of adipose-derived MSCs (Huang et al., 2023). Inflammatory preconditioning using cytokines such as IL-1β and TNF-α has also been studied to enhance MSC anti-inflammatory properties, preparing them to better tolerate the inflammatory microenvironment of degenerated discs (Le Maitre et al., 2005).

Chemical preconditioning with agents like lithium chloride has emerged as a promising approach. Lithium chloride enhances the proliferation and NP-like differentiation of ADMSCs, improving their adaptation to degenerative disc-like conditions by activating the ROS/ERK signaling axis, reducing cell death, and promoting ECM deposition (Zhu et al., 2021). While TGF-β3 is commonly used for chondrogenic differentiation, its effects on MSC survival under nutrient and oxygen deprivation are donor-dependent and may impair cell viability when used alone during monolayer expansion (Peck et al., 2021). These preconditioning strategies “arm” MSCs with enhanced survival mechanisms, improving therapeutic outcomes by helping them withstand the challenging conditions in the disc.

Among the currently described preconditioning strategies, hypoxic preconditioning currently appears to have the strongest evidence for improving both cell survival and therapeutic efficacy in DDD. Compared with other approaches, hypoxic preconditioning has shown more consistent benefits in enhancing implanted cell survival and migration, preserving disc height, and promoting matrix-related markers such as collagen II and ACAN *in vivo*, while also improving MSC survival under low-oxygen and nutrient-limited conditions. Other strategies, including quiescence, lithium, inflammatory stimulation, and mechanical stimulation, are also promising, but the current evidence remains more limited, model-dependent, or primarily focused on specific aspects such as differentiation or survival alone.

## 6 Application of MSCs in clinical trials for DDD

MSC transplantation has emerged as a promising therapeutic strategy for DDD, with encouraging safety and preliminary efficacy signals in early clinical studies of discogenic LBP. Initial trials generally report no serious procedure- or cell-related adverse events and clinically meaningful improvements in pain and function after intradiscal MSC-based interventions (Table 2).

**TABLE 2:** Characteristics and key outcomes of included clinical trials.

Study	Design	n	Cell product/Intervention	Follow-up	Key findings	References
Comella et al. (2017)	Single-arm prospective study	15	Autologous ADSC-containing SVF + PRP, intradiscal injection	6 months clinical; 12 months safety	No severe adverse events or infection were reported. Several pain and function measures improved; ODI and BDI trended in a favorable direction.	Comella et al. (2017)
Kumar et al. (2017)	Single-arm phase I trial	10	Autologous adipose-derived MSCs + HA, intradiscal injection	12 months	No procedure- or cell-related adverse events or serious adverse events were observed. Six of ten patients showed sustained clinical improvement; three showed increased disc water content on diffusion MRI.	Kumar et al. (2017)
Noriega et al. (2017)	Randomized controlled trial, sham-controlled	24	Allogeneic BM-MSCs, intradiscal injection	12 months	Feasibility and safety were confirmed. Pain and disability improved *versus* sham. MRI/Pfirrmann grade also improved; response appeared heterogeneous, with an apparent responder subgroup.	Noriega et al. (2017)
Centeno et al. (2017)	Registry-based pilot study	33	Culture-expanded autologous BM-MSCs, intradiscal injection	Up to 6 years	No serious adverse events were reported. Pain and function improved over long-term follow-up. Among patients with post-treatment MRI, 85% showed reduced disc bulge.	Centeno et al. (2017)
Blanco et al. (2019), Gomez-Ruiz et al. (2023)	Phase I/II, single-arm prospective trial	11	Autologous BM-MSCs embedded in tricalcium phosphate during posterolateral spinal fusion	5 years; 10 years	Long-term safety was maintained. Pain and disability improved. Fusion reached ∼80% at 5 years and all cases at 10 years; quality of life also improved.	(Blanco et al., 2019), (Gomez-Ruiz et al., 2023)
Lee et al. (2023)	Single-arm, open-label phase I trial	8	Matrilin-3-primed autologous ASC spheroids + HA, intradiscal injection	6 months	No procedure- or cell-related adverse events were reported. Six of eight patients met predefined VAS/ODI response criteria; radiological improvement was observed in four patients.	Lee et al. (2023)
Beall et al. (2025)	Multicenter, randomized, double-blind, concurrent-controlled trial	404	Allogeneic MPCs with or without HA vs. saline, intradiscal injection	36 months	The primary endpoint was not met in the full cohort. MPC + HA reduced mean pain *versus* control and showed stronger benefit in the prespecified subgroup with shorter pain duration; no treatment- or procedure-related serious adverse events were identified.	Beall et al. (2025)

In 2017, an open-label study evaluated intradiscal injection of stromal vascular fraction combined with platelet-rich plasma in 15 patients with DDD (Comella et al., 2017). Over 12 months, no severe adverse events were reported, and patients showed statistically significant improvements in flexion, VAS scores, present pain intensity, and short-form questionnaires. These findings support the feasibility and short-term safety of autologous adipose-derived cell therapy for disc degeneration. In the same year, a Phase I study assessed the safety of combined intradiscal ADMSCs and HA in 10 patients with chronic discogenic LBP (Kumar et al., 2017). During 12-month follow-up, no procedure- or cell-related adverse events were observed. Six patients demonstrated significant improvements in VAS, ODI, and SF-36 scores, and three showed increased disc water content on diffusion MRI, supporting that this approach is safe and well tolerated.

In a randomized controlled trial, Noriega et al. (2017) investigated intradiscal injection of allogeneic bone marrow MSCs in 24 patients with chronic discogenic pain. Compared with sham controls, the MSC group showed faster and greater improvements in algofunctional indices, and Pfirrmann grading suggested improved disc quality in treated patients. These data highlight the logistical advantages of allogeneic products and their potential to reduce pain and improve imaging outcomes. Long-term outcomes were reported by Centeno et al. (2017) in a registry-based study of 33 patients receiving autologous cultured bone marrow MSCs for DDD with radicular symptoms. Over 6 years, reductions in numeric pain scores and functional rating index scores were maintained, and MRI showed a reduction in disc bulge size in 85% of imaged patients. No serious adverse events were recorded, supporting durable symptomatic benefit and long-term safety in this cohort.

Blanco et al. (2019) and Gomez-Ruiz et al. (2023) reported a prospective phase I/II trial evaluating autologous BMSCs embedded in tricalcium phosphate for posterolateral spinal fusion in monosegmental lumbar DDD. Eleven patients with L4–L5 or L5–S1 degeneration refractory to conservative treatment received GMP-expanded autologous MSCs during instrumented posterolateral arthrodesis. At 5 years, the procedure was feasible and safe, with no implantation-related adverse effects, radiologic fusion in 80% of patients, and significant improvements in VAS and ODI scores. At 10 years, long-term safety was confirmed, with no tumor formation, infection, or heterotopic ossification; radiologic fusion reached 100%, and clinical improvements were sustained over the decade. A Phase I trial also evaluated matrilin-3-primed ADMSC spheroids plus HA in eight patients with chronic discogenic LBP (Lee et al., 2023). At 6 months, six patients met success criteria (≥2-point VAS reduction and ≥10-point ODI improvement), with radiologic improvement in half of responders and no treatment-related adverse events, supporting feasibility and safety of functionally enhanced MSC spheroids.

Most recently, a prospective, multicenter, randomized, double-blind, concurrent-controlled 36-month study (Beall et al., 2025) evaluated a single intradiscal injection of allogeneic MSCs, with or without HA, in 404 patients with chronic LBP associated with moderate DDD (Beall et al., 2025). Although the primary composite endpoint was not met, the MPC + HA arm showed statistically significant reductions in least-squares mean VAS scores *versus* saline at 12 and 24 months in the overall cohort. In a prespecified subgroup with symptom duration <68 months, MPC + HA produced greater pain reduction across all time points through 36 months, with improved function and quality of life. A higher proportion of baseline opioid users in the MPC + HA group discontinued opioids by 36 months, and treatment was well tolerated, with no serious procedure- or product-related adverse events.

Collectively, these studies support the short-to mid-term safety of MSC-based interventions for discogenic pain and suggest potential benefits in pain, function, and—selectively—imaging outcomes. Moving forward, adherence to standardized clinical protocols (cell source, dose, delivery approach, imaging endpoints, and harmonized outcome measures) will be essential to validate efficacy and enable translation of MSC therapies into routine practice for disc regeneration.

## 7 Challenges in clinical application and future directions

The clinical translation of MSC- and Exo-based therapies for DDD remains constrained by three interrelated barriers: manufacturing standardization, regulatory approval, and cost (Jayaraman et al., 2021; Strecanska et al., 2025). Product standardization remains a central obstacle. Variability in donor characteristics, tissue source, MSC expansion protocols, culture conditions, and Exo isolation methods can substantially alter product quality and therapeutic performance. Current Exo isolation approaches, including ultracentrifugation, size-based filtration, and affinity capture, often generate marked heterogeneity in vesicle yield, purity, and cargo composition. Likewise, differences in MSC procurement and expansion can affect cell identity, viability, and functional stability. GMP-compliant manufacturing is therefore foundational rather than optional. For MSC-based products, this requires standardized donor screening, master and working cell bank systems, and closed, automated expansion platforms that preserve critical product attributes during scale-up. At the current stage, one of the most realistic translational routes is to prioritize banked, allogeneic, off-the-shelf MSC products, because such platforms are more compatible with reproducible batch manufacturing and large-scale distribution than patient-specific autologous approaches. For Exo-based products, translation will depend on integrated and scalable upstream–downstream workflows that combine high-yield bioreactor culture with standardized purification suitable for large-batch production, storage, and release testing.

Within this framework, rigorous quality control is indispensable. Assays for identity, purity, and batch-to-batch consistency are required for both MSC and Exo products (De Wolf et al., 2017). For Exos, particular attention should be given to purity and cargo consistency, including proteins, RNA, and lipids. Potency testing is especially important because it links manufacturing control to regulatory evaluation. In DDD, potency assays should be mechanism-linked and aligned with the intended therapeutic actions of the product, especially anti-inflammatory activity and extracellular matrix preservation or regeneration. A practical near-term step is to establish mechanism-linked potency assays early in development and incorporate them into process optimization, batch release, and comparability assessment, rather than treating potency testing only as a late-stage regulatory requirement. Mechanism-linked potency assays would provide a biologically relevant basis for batch release, comparability assessment, and cross-center consistency testing.

Regulatory approval remains another major barrier, particularly for cell-based products. MSC- and Exo-based therapies require robust evidence of safety and efficacy before clinical use (Zhao et al., 2021). Immunogenicity remains a concern, especially for allogeneic MSCs, and long-term safety must be carefully assessed. These issues require staged preclinical testing and well-designed clinical trials with adequate follow-up. Early regulatory interaction through pre-IND, pre-CTA, or scientific advice pathways is therefore one of the most realistic steps to facilitate translation. Because it can clarify product classification, chemistry, manufacturing and controls (CMC) requirements, potency strategy, and clinically meaningful endpoint selection before late-stage development. For DDD, endpoint design should capture not only general safety and efficacy, but also disease-relevant outcomes such as pain relief, functional improvement, and imaging-based structural measures. Early clinical programs may also benefit from narrower patient stratification and harmonized outcome sets, which could improve trial interpretability and reduce translational uncertainty.

Economic barriers are also substantial. The production of MSCs and Exos is resource-intensive, with major costs arising from GMP compliance, process control, and manufacturing scale-up (Stawarska et al., 2024). Preclinical development, clinical testing, and long-term follow-up further increase the financial burden (Rejon-Parrilla et al., 2023). More efficient manufacturing strategies will therefore be necessary to improve accessibility. Standardized allogeneic MSC or Exo products are more compatible with broad clinical deployment than patient-specific autologous approaches, because they shift production from a one-patient/one-batch model to a one-batch/multi-patient model. This can reduce per-patient cost, simplify quality control, improve scheduling flexibility under GMP conditions, and better support commercial-scale supply. Cost reduction, however, should not be viewed solely as a manufacturing issue. Closed-system processing, automation-compatible expansion, scalable bioreactor culture, and standardized purification can reduce labor demand, contamination risk, batch failure, and inter-site variability during scale-up. At the same time, health economic planning should begin during early development rather than after approval. Without evidence of cost-effectiveness, budget impact, and reimbursement feasibility, broad uptake is likely to remain limited even for approved products.

Taken together, several near-term priorities may facilitate the clinical translation of MSC- and Exo-based therapies for DDD. These include industrialized GMP-compliant manufacturing, mechanism-linked potency frameworks aligned with DDD-relevant therapeutic functions, early regulatory engagement through pre-IND or pre-CTA pathways, and the integration of health economic considerations into development strategy. More specifically, the most realistic next steps for large-scale clinical translation are to prioritize off-the-shelf allogeneic product platforms, define potency and critical quality frameworks early, align CMC strategy and clinical endpoints with regulators before late-stage development, and incorporate reimbursement and market-access planning during early translational development. These measures directly address current constraints in reproducibility, approval, affordability, and accessibility, and may provide a practical foundation for future therapeutic refinement.

Looking ahead, future development is likely to extend beyond these immediate translational priorities. Personalized approaches may improve treatment matching by incorporating patient-specific biological features, including inflammatory or genetic profiles, to guide source selection or preconditioning strategies. Advanced biomaterials may further improve the local retention and functional persistence of MSC- or Exo-based products within the hostile disc microenvironment. In parallel, IVD organoids and 3D bioprinting may provide useful platforms for disease modelling, drug screening, and the design of anatomically relevant scaffold-based implants. Cell-free strategies are also likely to expand. Exo-loaded biomaterials may enable sustained local delivery of therapeutic signals in the degenerative disc environment. Engineered Exos, including vesicles modified with targeting ligands or loaded with defined growth factors or RNA cargos, may further enhance tissue specificity and therapeutic precision. At present, however, these approaches should be regarded as emerging directions rather than immediate clinical solutions. Their eventual translational value will depend on whether they can be integrated into manufacturable, regulatable, and economically viable product frameworks.

## 8 Conclusion

MSC and Exo therapies offer significant promise for transforming DDD management by providing minimally invasive, regenerative strategies that address the disease’s underlying pathology, rather than merely alleviating symptoms. MSCs and their derived Exos have shown considerable potential in promoting tissue regeneration, reducing inflammation, and restoring disc function. These therapies aim to rejuvenate the IVD by delivering regenerative signals directly to the affected area, offering a novel approach to DDD treatment. Unlike traditional surgical interventions, these biologics target the root cause of disc degeneration, offering a viable pathway toward long-term relief and improved quality of life for patients. Their potential for providing regenerative, less invasive options makes them a compelling alternative to conventional treatments. However, to fully realize their potential, long-term research, improved delivery methods, and robust regulatory frameworks are essential. Addressing these challenges will be crucial for translating the promise of these therapies into tangible clinical benefits.
